# The structure of the antimicrobial human cathelicidin LL-37 shows oligomerization and channel formation in the presence of membrane mimics

**DOI:** 10.1038/s41598-020-74401-5

**Published:** 2020-10-15

**Authors:** Enea Sancho-Vaello, David Gil-Carton, Patrice François, Eve-Julie Bonetti, Mohamed Kreir, Karunakar Reddy Pothula, Ulrich Kleinekathöfer, Kornelius Zeth

**Affiliations:** 1 Unidad de Biofisica, Centro Mixto Consejo Superior de Investigaciones Cientificas-Universidad del País Vasco/Euskal Herriko Unibertsitatea (CSIC, UPV/EHU), Barrio Sarriena s/n, Leioa, Bizkaia Spain; 2 Structural Biology Unit, CIC bioGUNE, Parque Tecnológico de Bizkaia Edificio 800, 48160 Derio, Spain; 3 Genomic Research Laboratory, Department of Medical Specialities, Geneva University Hospitals, University of Geneva, Genève, Switzerland; 4 Nanion Technologies GmbH, Gabrielenstraße 9, 80636 Munich, Germany; 5 Department of Physics and Earth Sciences, Jacobs University Bremen, Campus Ring 1, 28759 Bremen, Germany; 6 Department of Science and Environment, Roskilde University, Universitetsvej 1, 4000 Roskilde, Denmark; 7 Present Address: Institute of Microbiology and Infection, College of Medical and Dental Sciences, University of Birmingham, Edgbaston, Birmingham, UK; 8 Present Address: Janssen Pharmaceutica NV, Janssen R&D, Nonclinical Safety, Beerse, Belgium

**Keywords:** Peptides, Membrane biophysics, Biophysical chemistry, Mechanism of action, X-ray crystallography

## Abstract

The human cathelicidin LL-37 serves a critical role in the innate immune system defending bacterial infections. LL-37 can interact with molecules of the cell wall and perforate cytoplasmic membranes resulting in bacterial cell death. To test the interactions of LL-37 and bacterial cell wall components we crystallized LL-37 in the presence of detergents and obtained the structure of a narrow tetrameric channel with a strongly charged core. The formation of a tetramer was further studied by cross-linking in the presence of detergents and lipids. Using planar lipid membranes a small but defined conductivity of this channel could be demonstrated. Molecular dynamic simulations underline the stability of this channel in membranes and demonstrate pathways for the passage of water molecules. Time lapse studies of *E. coli* cells treated with LL-37 show membrane discontinuities in the outer membrane followed by cell wall damage and cell death. Collectively, our results open a venue to the understanding of a novel AMP killing mechanism and allows the rational design of LL-37 derivatives with enhanced bactericidal activity.

## Introduction

The increase in antibiotic resistance is one of the biggest health challenges our society is currently facing^1^. As a consequence, the discovery of new bactericidal drug candidates from any source including antimicrobial peptides (AMPs) is urgent^2–4^. According to recent studies antimicrobial peptides have the potential to become lead structures due to their ability to effectively eradicate pathogenic bacteria and biofilms^5–7^. However, in spite of the large number of AMPs only a minor fraction was introduced into clinical studies and none of those went into the pharmaceutical market^8^. This failure converting natural AMPs to drug candidates can be traced to our lack of understanding the mechanisms whereby they target and kill bacterial cells, hence the rational basis for a design of peptides with tailored properties is impeded. Therefore more AMP structures, ideally in complex with their cellular targets, are needed to track their pathways and define interfaces of AMP/target-complexes. Using this information, we will be able to specifically enhance their properties by peptide design^9^.

AMPs in mammals form part of the innate immune response^4^. Their activity includes functions in microbial killing, inflammation, angiogenesis and wound healing^4,10^. According to recent studies the physiological concentrations of some AMPs may be sufficient to densely cover the bacterial surface and initiate bacterial cell death^11^. Most AMPs typically carry a significant surplus of surface exposed positive charges and hence, they can initially interact with the negatively charged cell surface exposed lipopolysaccharides (LPS) or lipoteichoic acid (LTA) molecules of Gram-negative and -positive bacteria eventually extracting these molecules from the cell wall before entering the cell^12–15^**.** Further steps of action depend on the peptide under investigation and the bacterial cell type. It is assumed that amphipathic and α-helical AMPs can perforate the cytoplasmic membrane, a structure also considered to be the ‘Achilles heel of bacterial cells’, resulting in the breakdown of the transmembrane potential^3^. Due to the structural variability of AMPs and their unspecific mode of action various interaction partners (either specific or unspecific) are known at the cell wall, the periplasmic space and inside the bacterial cytoplasm^16–20^. Within the cytoplasm some AMPs can target protein, DNA or RNA complexes such as the ribosome^21,22^. As an example, the complex of the ribosome-associated Onc112 peptide and the *Thermus thermophilus* 70S ribosome has been discovered and provided the first structural information of an AMP at high resolution with its specific target^21,22^.

One of the first steps of the cellular targeting may include binding and oligomerization of AMPs at cell wall constituents (LPS, LTA, lipid membranes). Oligomerization and fiber formation of AMPs has recently been shown to occur on lipid vesicles and can also be initiated by detergents, a mechanism possibly important for AMP activation and killing^23,24^. One of the first studies describing fiber formation via end-to-end alignment was based on the fluorescently labelled cationic peptide LAH4 when attached to membranes^25^. In addition, supramolecular structures based on their higher oligomeric state in crystal lattices were deduced from the BTD-2 antimicrobial peptide or the membrane-disruptive phenol-soluble modulin alpha 3 (PSMα3)^26–28^. BTD-2 peptides organize in a fibril-like state within the crystal lattice similar to fibrils of PSMα3, both of which appear to form amyloid-like states via zipper motifs providing structural evidence linking antimicrobial and amyloid peptides^27^. LL-37 has also been shown to form fibres when incubated with lipid vesicles using electron microscopy^23^. In our own study we showed the supramolecular formation of LL-37 fibers in crystal lattices mediated in an head-to-tail arrangement of dimers. We confirmed these fibers by electron microscopy using gold-labelled peptides incubated with lipid vesicles^24^.

Membrane perturbation is considered to be another, possibly subsequent mechanistic step during the course of cellular targeting. Three simple mechanistic models depict this interaction of α-helical amphipathic AMPs with uniform bilayers. However, these models are insufficient in their ability to explain the biological context of AMP-membrane interactions under in vivo conditions due to their simplicity^9,12,29,30^. The models describe interactions with artificial lipid membranes on the basis of moderate to strong hydrophobic and hydrophilic attraction within peptide-lipid complexes, initiating membrane disturbance. The barrel-stave model emphasizes the assembly of peptides leading to oligomeric transmembrane channels which expose their hydrophobic surfaces towards artificial membranes^31^. The toroidal model describes the pore formation by AMPs strongly interacting with lipid head groups and alkyl chains but assuming only weak peptide-peptide interactions^32,33^. Finally, the carpet model explains the disintegration of the artificial membrane in a detergent-like manner, with AMPs encapsulating lipid micelles^34^.

The barrel stave model which describes a rigid membrane-disrupting peptide complex is possibly the most detailed data-based mechanistic model. However, up until now, there is only one AMP structure of Dermcidin (DCD) published by our laboratory available which demonstrates a defined transmembrane channel validating the barrel stave model for this peptide^35^. DCD shows an unusual strongly charged channel of 8 nm length exhibiting discrete conductance steps in planar membranes^9,35,36^. Although the structure was solved in the absence of detergents and lipids, the channel clearly shows the archetypical features of six membrane-spanning helices arranged in pairs of anti-parallel dimers with a hydrophobic surface facing the putative membrane. Another channel-forming AMP is alamethicin, a well-studied peptide that has served as a paradigm for voltage-gated channels. The peptide was probed by Woolley to be a dimer^37^ but also a hexameric channel structure was proposed^38,39^. The hexameric state of alamethicin is supported by another study on using electrochemical scanning tunnelling microscopy (EC-STM) which shows hexameric pores formed in a matrix of phospholipids^40^.

Human cathelicidin comprises the cathelin precursor domain and LL-37 as C-terminal extension^41,42^. LL-37 is activated through proteolytic cleavage as a mature peptide^43^ comprising 37 amino acids including two N-terminal leucines and a net positive charge of + 6. This peptide was extensively studied by biochemical, biophysical and structural methods^10^. Early studies by NMR on LL-37 dissolved in a buffer comprising the denaturing SDS showed a monomeric and strongly bent helical structure^44^. By contrast, recent crystallographic studies demonstrated an anti-parallel dimer of peptides in the absence of detergents^24^. This dimer undergoes significant structural changes in the N- and C-terminus after interacting with detergents such as dodecylphosphocholine (DPC) or lauryldimethylamine oxide (LDAO), and defined and stable detergent interaction sites were obtained in high resolution structures likely resembling lipid binding sites in vivo. These sites are also observed by NMR of LL-37 in the presence of DPC proving the same interacting aromatic residues mapped on a tripartite structure^45^.

The mechanism by which LL-37 interacts with the bacterial cell wall has not been investigated in much detail. Consequently, our primary aim was to understand how LL-37 interacts with molecules of the bacterial cell wall and to characterize these interactions based on secondary, tertiary and quaternary structures, respectively. Here we report crystallographic investigations which reveal the tetrameric structure of LL-37 crystallized in the presence of DPC. The existence of this oligomer in lipid bilayers was proven by cross-linking and circular dichroism (CD). Molecular dynamic studies of the tetramer embedded in a bacterial membrane system proved physical stability and flow of water molecules through the channel was monitored. To investigate the function of this channel structure we used planar lipid membranes and determined a small but defined conductivity. Finally, in vivo studies using electron microsopy of *E. coli* cells incubated with LL-37 show discontinuities in the outer and inner membrane followed by the breakdown of the membrane system. Together with a previous paper on the fiber formation of LL-37 our data form the basis to construct a new mechanism of LL-37 targeting bacterial cells. Together these results allow for the rational design of LL-37-derived peptides as potential peptide-based antibiotics.

## Results

### LL-37 forms a narrow tetrameric channel structure in the presence of DPC

**Figure 1:**
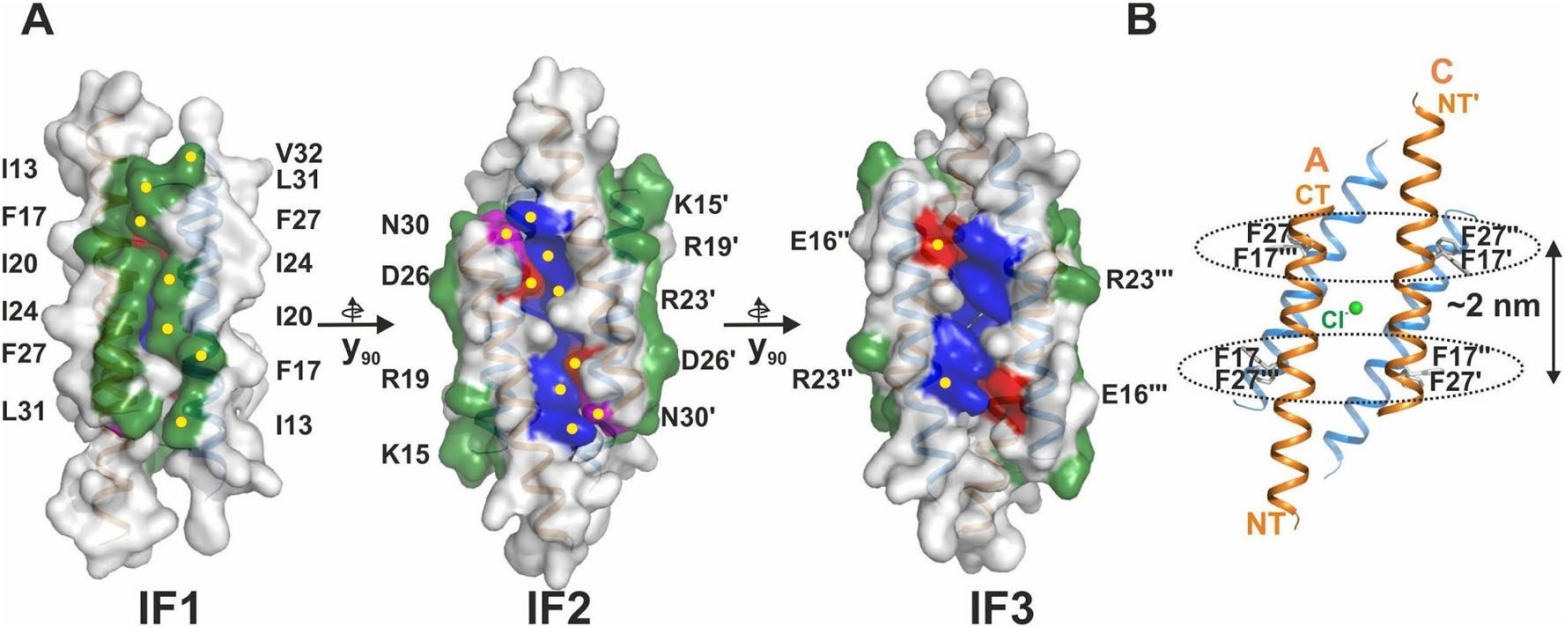
The structure of LL-37_DPC-4_ provides insights into the putative transmembrane channel architecture. **(A)** LL-37_DPC-4_ in surface representation and analysis of IF1-IF3 viewing the side dominated by hydrophobic residues emphasized in green and marked by sequence numbers. The IF1 is formed by hydrophobic residues, while IF2 and IF3 are formed by a mix of polar and charged residues (marked magenta, red and blue surface). Residues involved in the maintenance of each interface are marked by numbers. **(B)** Aromatic girdles in the tetrameric arrangement indicate the potential for membrane integration architecture. Two phenylalanines of each peptide (Phe17 and Phe27) contribute to the formation of these regular girdles separated by a distance of ~ 1.5 nm.

We crystallized LL-37 in the presence of detergents to achieve new detergent-induced structural states of LL-37 states. Previously we obtained peptide-detergent complexes comprising structurally ordered detergents attached to hydrophobic surface areas (structure termed LL-37_DPC-2_ in the following). In our new crystallization attempts of LL-37 in 1% DPC crystals were formed in the presence of 70% MPD. The structure was solved by molecular replacement using the monomer structure of LL-37_DPC-2_ as a search model. Two antiparallel monomers in the asymmetric unit are crystallographically related to a second dimer, together building a narrow tetrameric 4 nm long channel structure (termed LL-37_DPC-4_; see Figs. 1A, S1D, S2). Notably, this channel does not follow an exact twofold symmetry along the crystallographic channel axis (see Figs. 1, S1D, S2). Disordered residues are observed at both termini resulting in an ordered channel core structure only comprising residues Phe6 to Asn30 (see Figs. 1, S1). Although narrow, the channel shows a continuous and charged inner cavity between the terminal pores with a constriction at the centre (see Fig. S2B). Due to this asymmetric arrangement three different peptide interfaces (IF1, IF2 and IF3) are formed, two of which (IF1) are identical dimer interfaces stabilized by the hydrophobic residues Ile13, Phe17, Ile20, Ile24, Phe27 and Leu31 (interface area 445 Å^2^—see Fig. 1A). IF1 is further stabilized by two salt bridges involving residues Glu16 and Arg23 (see Fig. S2C). The IF2 is stabilized by four hydrophilic residue pairs Lys15/Asn30 and Arg19/Asp26 (contact area 344 Å^2^; see Fig. S2C). In analogy to IF2 also the smallest interface IF3 is formed by four salt bridges including residues Glu16/Arg23 and Arg19/Asp26 (contact area 339 Å^2^; see Figs. 1A, S2C). In the interior of the channel-like structure a chloride ion is trapped by two Arg23 residues from adjacent monomers and further coordinated by two water molecules (see Figs. 1B, S1, S2C). The interior of the channel displays a dense network of H-bond interactions and 15 coordinated water molecules while water molecules surrounding the tetrameric channel are essentially absent (see Fig. S1D). The charged channel core, with a surplus of positive charges and 3 nm in length, is delimited by two aromatic girdles (formed by eight Phe residues, four Phe17 and four Phe27 residues) arranged at a distance of ~ 2 nm pointing towards the putative membrane environment, respectively (see Fig. 1B).

We analyzed all crystallographically determined LL-37 structures of this and a previous paper to identify structural changes in response to the presence of detergents as well as the monomer and dimer conformations (see Fig. S3)^24^. Firstly, the superposition of the two peptides in the asymmetric unit of LL-37_DPC-4_ proves them to be identical with a r.m.s.d of 0.4 Å (for CA atoms of residues 6–30). Interestingly, the dimer architecture of this structure is very different to the previously reported structures of LL-37_DPC-2_ and the structure crystallized in the absence of detergents LL-37^24^ (see Fig. S3). The two monomers of the LL-37_DPC-4_ asymmetric unit need to be shifted by about five turns relative to each other to form the LL-37_DPC-2_ structure. Secondly, the r.m.s.d of the monomer in LL-37_DPC-4_ relative to LL-37 structure (crystallized without detergents) is 1.2 Å while the deviation to the LL-37_DPC-2_ structure monomer is only 0.4 Å, indicating a better structural fit between the detergent induced structures. In summary, the tetrameric structure of LL-37 comprising antiparallel peptide monomers is formed on the basis of three different hydrophobic or hydrophilic interfaces, respectively.

### Studies on the secondary structure and oligomerization state of LL-37 upon interaction with detergent and lipids confirm the existence of a tetramer

In order to show that the crystallized state induced by detergents mimics a structural state found in lipid membranes, we monitored secondary structure and oligomeric state changes in response to detergents and lipids. First, LL-37/DPC mixtures were employed in physiological buffer at increasing detergent concentrations with a starting concentration below the critical micelle concentration (CMC) (100 mM NaCl, 5 mM phosphate, pH 7; 0.14 mM—5.7 mM, CMC: 1.5 mM). The spectrum obtained for LL-37 alone displays high ellipticity values at 208 and 222 nm, typical for α-helical structures. Because LL-37 is a well folded peptide in a buffer containing 100 mM NaCl, the intensity of the spectrum changed only marginally in response to increasing DPC concentrations (see Fig. S4). Cross-linking in the presence of 0.2–1% DPC using glutaraldehyde and BS3 as a cross-linker yielded monomeric-tetrameric bands at decreasing intensity (data not shown).

**Figure 2:**
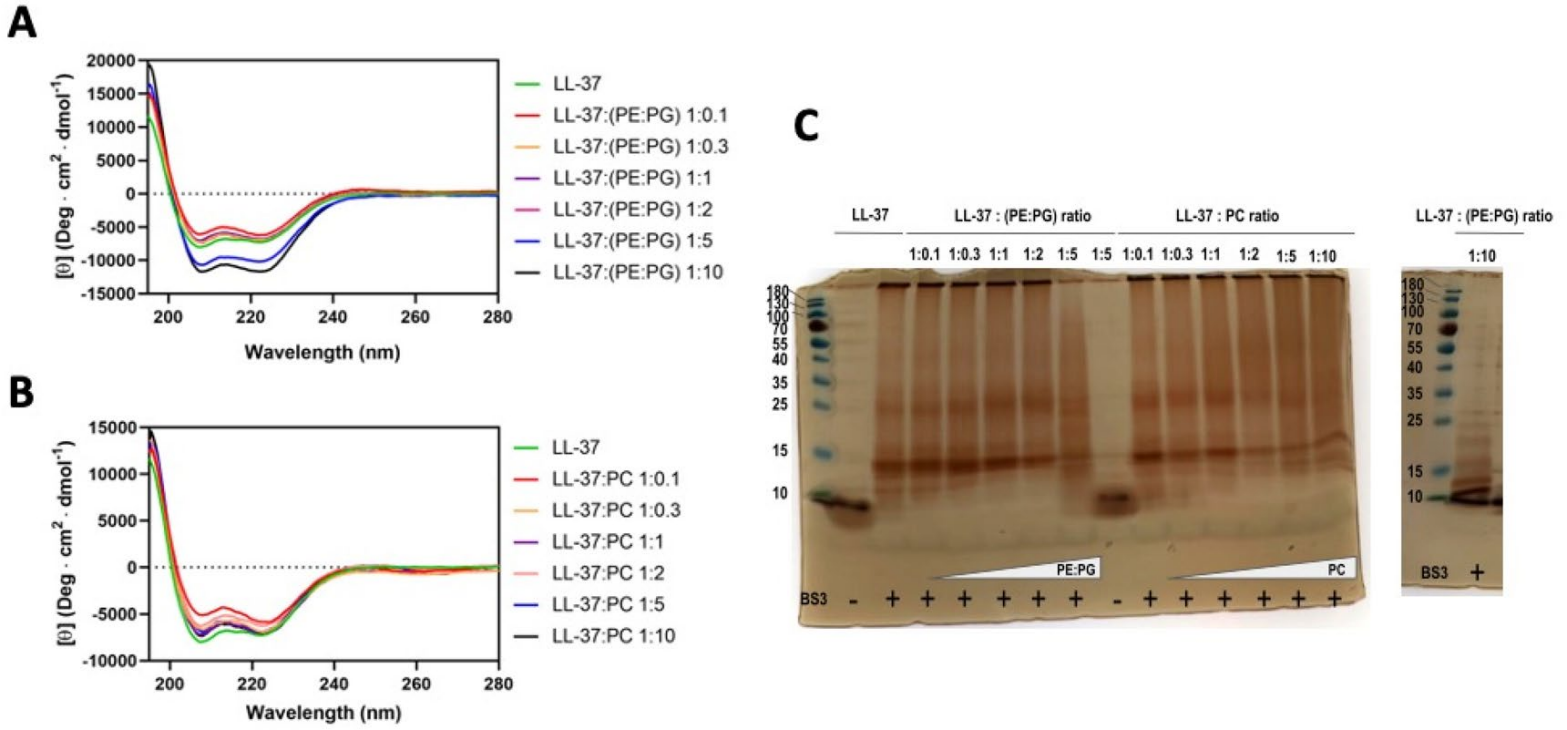
Analysis of the folding and oligomerization states of LL-37 in the presence of PE:PG (3:1) or PC by circular dichroism and cross-linking. **(A)** Circular dichroism at different LL-37/(PE:PG) ratios. Only at higher LL-37:(PE:PG) ratios (see blue and black lines for 1:5 and 1:10 ratios, respectively) did the ellipticity relative to the spectrum in the absence of lipids increase. **(B)** Circular dichroism of LL-37/PC mixtures. At the smallest LL-37:PC ratio (see red line for 1:0.1 ratio) a small unfolding of LL-37 is suggested. **(C)** Cross-linking of LL-37 in the presence of the mixture PE:PG (3:1) or PC using BS3 as a cross-linker. Left gel: Lane 1: Molecular weight marker; Lanes 2–3: LL-37 in the absence and presence of BS3; Lanes 4–8: LL-37/(PE:PG) mixture at 1:0.1, 1:0.3, 1:1, 1:2 and 1:5 ratios; Lane 9: same as lane 8 but lacking BS3; Lanes 10–15: LL-37/PC mixtures at 1:0.1, 1:0.3, 1:1, 1:2, 1:5 and 1:10 ratios. Right gel: Lane 1: Molecular weight marker; Lane 2: LL-37/(PE:PG) mixtures at 1:10. The gels were silver stained.

In order to prove the existence of the LL-37 tetramer in membrane-like environments, we performed studies on the secondary structure and oligomerization behavior in the presence of lipids. Since phosphatidylethanolamine (PE) and phosphatidylglycerol (PG) are the main lipid components of the inner bacterial membrane a mixture of PE:PG (3:1) was employed for these experiments^46^. To compare the effect of another non-bacterial lipid, we chose phosphatidylcholine (PC) which is the major component of eukaryotic cell membranes and has the same head group as DPC^47^*.* At high ratios of LL-37:PE/PG, the ellipticity increased significantly while the secondary structure remained essentially the same for all LL-37:PC ratios, resembling the results from DPC measurements (see Fig. 2A,B). In the presence of all the tested lipids, the typical α-helix spectrum with the minimum at 208 and 222 nm confirmed the expected folding for the peptide. In order to characterize the oligomerization state of LL-37 under the same conditions chosen for CD, we performed cross-linking experiments using BS3 as a cross-linker. In the absence of lipids and BS3, a dimer of LL-37 (at ~ 9 kD) was observed. After adding BS3 alone, a strong band corresponding to tetramers (at ~ 15 kD) was observed with a weak band in between potentially representing trimers. In the presence of BS3 and lipids, the most prominent band corresponds to the tetramer and a weak band corresponding to a hexamer at ~ 25 kD is visible at higher LL-37:lipid ratios (see Fig. 2C). Trimer and tetramer bands only disappeared at a high PE:PG ratio of 1:5 but remained at the same ratio for the LL-37:PC mixture. These experiments support the existence and stabilization of a LL-37 tetramer in the presence of both bacterial and eukaryotic membrane lipids particularly at higher lipid:peptide ratios.

Based on the crystal structures we designed two points mutants (E16A and R23A) to decipher the influence of single residues onto the structure conformation, oligomerization and activity of LL-37. The mutant residues are the main interacting residues at the dimer interface. Here, Glu16 forms a salt bridge with Lys12 and a hydrogen bond with Ser9 in the LL-37 structure in the dimeric LL-37 structure (PDB:5NNM). In the LL-37_DPC-2_ structure (PDB:5NNT) crystallized in the presence of DPC, Glu16 interacts with Ser9 but not Lys12. Arg23 has strong implications in the formation of the fibrils as introduced in the paper by Sancho-Vaello et al. and interacts with the head group of the detergents DPC and LDAO used for crystallization*.* In LL-37_DPC-4_ the Glu16 residue interacts with three positively charged residues Lys12, Arg19 and Arg23 in the first peptide chain as well as Lys12 and Arg23 but not Arg19 in the second. In CD, only E16A exhibited a decrease in ellipticity, which was recovered after addition of detergent (see Fig. S5A). The pattern of bands in the cross-linking using BS3 was altered for both mutants. Whereas E16A does not show any evidence of oligomerization and seemed to have precipitated under the chosen conditions (reproducible loss of signal in the gel), R23A showed oligomerization but different to the one exhibited by the wild type peptide (see Fig. S5B).

### LL-37 forms ohmic conductance pores in artificial planar membranes

**Figure 3:**
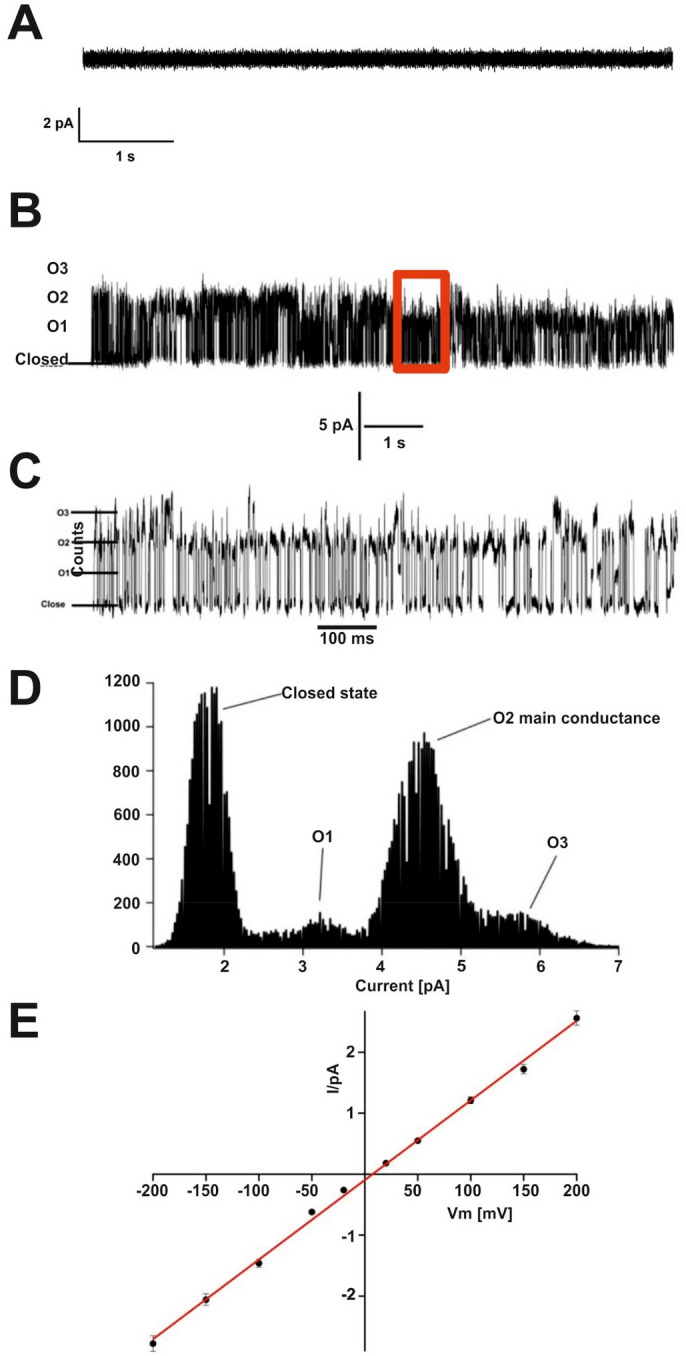
Conductivity determination of LL-37 in planar lipid membranes. **(A)** No-peptide control. This trace was recorded before any addition of peptide. **(B)** Recording of LL-37 channel activity in planar lipid bilayers at 200 mM KCl, 10 mM HEPES, pH 7 inside and 4 outside. The channel characteristics were recorded for several seconds and demonstrate the rapid conductivity fluctuations of the channel in lipid membranes. The scale bar (5 pA) below the conductivity trace indicates the height of conductivity events. **(C)** Zoom in of the section marked with a red box in the channel recording of (A) depicted in another time scale indicates the fast conductance events in a 100 ms range (see scale bar). The current profile indicates three recurring conductivities termed O1, O2 and O3. **(D)** Channel conductances shown in (B) are quantified and current events (counts) were plotted against the current values in picoamperes (pA). This graph shows O2 to be the major conductance state with 13 pA, while additional O1 and O3 are recognized. **(E)** Current versus voltage graph for LL-37 at − 200 to + 200 mV. The linear progression of conductivity with increasing voltage from − 200 to + 200 mV shows an ohmic behavior of the channel in planar lipid membranes.

To obtain additional functional evidence for the formation of a channel and its activity in artificial membranes we determined conductivity in planar lipid bilayers (the control line was recorded before any addition of peptide, see Fig. 3A). Membranes containing 1,2-diphytanoyl-sn-glycero-3-phosphocholine (DPhPC) and cholesterol were prepared and LL-37 was applied in concentrations similar to those used in antimicrobial assays. The voltage-conductivity relationship of the channel was determined to be linear between -200 and + 200 mV following an ohmic behavior (Fig. 3E). Transmembrane current steps at 200 mM KCl were observed from which three main conductances of Closed state (C) to O1 = 13.3 pS, O1 to O2 = 12.8 pS and O2 to O3 = 12.96 pS could be distinguished (see Fig. 3B–D). The conductances were calculated for the different levels of openings using a Gaussian fit for all the points in the histogram^48^. The major conductance step was determined to be O2 with 900 counted states in comparison to O1 and O3 with approximately 100 signals counted (see Fig. 3D). These mean openings allowed us to calculate the average channel conductance of 13 pS at a voltage of 100 mV with a mean open lifetime that was estimated to be τ = 1.8 ms and an open probability *P*_*O*_ of 0.71 (see Fig. S6). In summary, the planar lipid membrane measurements confirm the formation of a LL-37 peptide channel with a defined but small conductance.

### Molecular dynamic simulations demonstrate channel stability in POPE/POPG membranes

**Figure 4:**
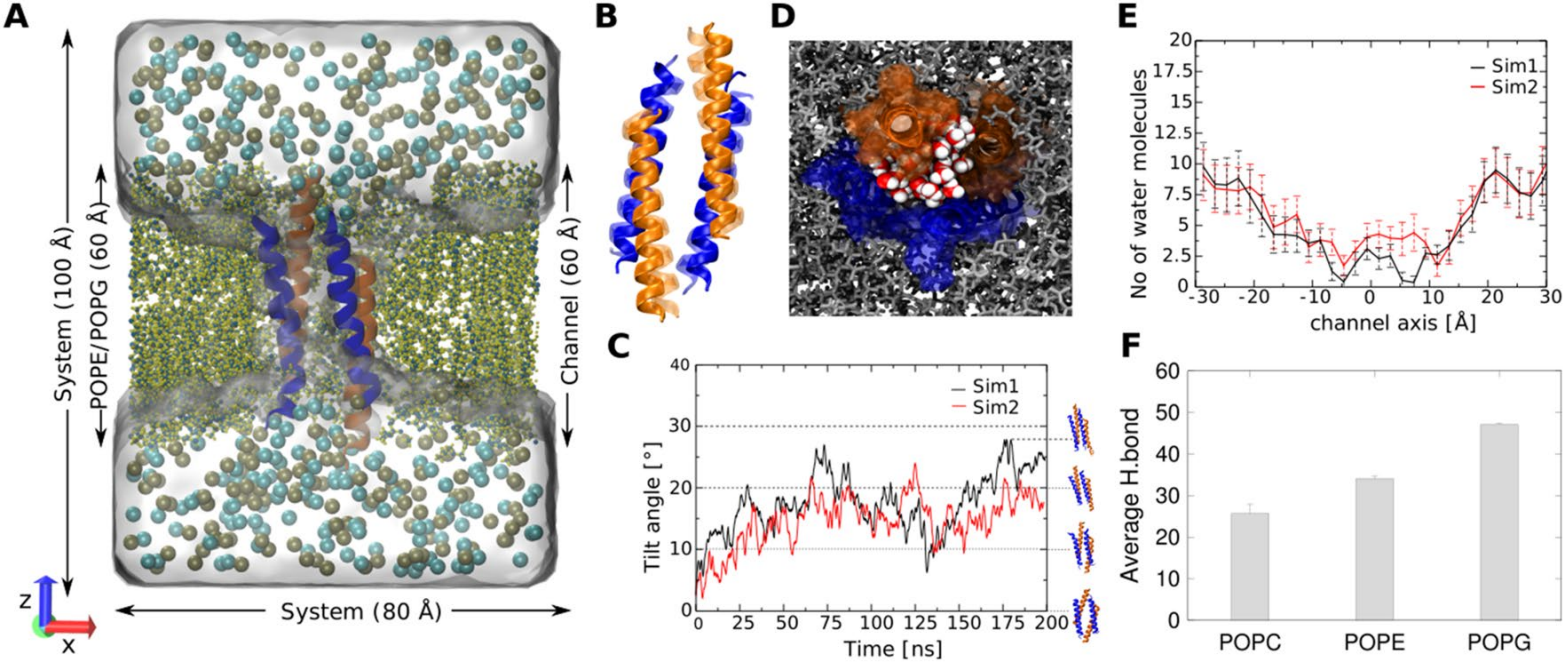
Molecular dynamics studies model the channel behaviour in membranes. **(A) **System setup used in the MD simulations. The LL-37 channel (orange and deep blue helices) embedded in a POPE/POPG (3:1) bilayer membrane (yellow and cyan) and a 1 M KCl salt solution (K^+^: cyan spheres, Cl^−^: brown spheres). For clarity the front part of the lipids has been omitted in the figure. **(B)** Side view of the comparison between the crystal structure and the average structure calculated from the MD trajectories. The crystal structure conformation is shown in a transparent fashion. **(C)** Change in the orientation (measured using the tilt angle) of the LL-37 channel during the simulations in POPE/POPG bilayer and their respective snapshots. **(D)** Water distribution (red/white spheres) in the LL-37 channel in the lipid bilayer (gray stick representation). **(E)** Average number of water molecules in bins of 2 Å width inside the channel averaged over the last 100 ns trajectory from each simulation. **(F)** Average number of hydrogen bonds formed by LL-37 with the respective membrane bilayer.

In order to examine if the tetrameric structure was energetically stable when embedded in artificial membranes, MD simulations of a peptide-membrane system were executed. Unbiased simulations of the channel in a phosphatidyl ethanolamine/phosphatidyl glycerol (POPE/POPG, 3:1) bilayer were performed (see Fig. 4A). As shown in Fig. 4B, the LL-37_DPC-4_ structure embedded in membranes does not vary considerably during the simulations. The various MD states show r.m.s.d values of 2.5 Å to 3 Å including all Cα-atoms relative to the crystals structure (see Fig. S7A). To further examine the structural stability we also monitored the interhelical distances to investigate the packing arrangement relative to the crystal structure. The interhelical distances of adjacent helix pairs remained largely undisturbed for the time of simulation indicating no significant changes, and leading to the conclusion that the structure is inherently stable under the conditions chosen (see Fig. S7B).

To determine the in-membrane movement of LL-37, including the tilt angle between the channel axis and the bilayer normal, we performed 200 ns simulations towards equilibrium states. These states normally reflect the peptide-membrane architecture at an energetic minimum after matching hydrophobic and hydrophilic properties of peptides and lipid chemistry. In two independent simulations, the channel was found to be tilted by about 20 degrees with respect to the membrane (see Fig. 4C). The energetic minimum matches the hydrophobic length of the channel with the membrane thickness and positively charged residues on the external interface with negatively charged POPG residues in the membrane.

When water molecules were added to the system they rapidly occupied positions in the interior of the asymmetric peptide channel during unbiased simulations and their localization is shown in Fig. 4D. The water distribution in the different slices along the channel axis is shown in Fig. 4E. Furthermore, we noticed a preferential movement of waters along IF2 and IF3 which is in agreement with the water distribution in the crystal structure (Fig. S8 for the water distribution in the channel). Furthermore, we tested the stability of the tetramer channel in single component bilayers (POPC, POPE and POPG) and found that the structure showed similar rmsd changes (Fig. S7C) and tilt angles (Fig. S7D) as that of the structure in a multiple component POPE/POPG bilayer. Consistent with the fact that LL-37 has a higher selectivity for prokaryotic than for eukaryotic membranes, in simulations LL-37 shows fewer hydrogen bond interactions with the POPC membrane than with the POPE and POPG membranes wherein stable protein-lipid complexes are formed (Fig. 4F). Collectively, we can demonstrate that LL-37 is stable during all MD simulations in POPE and POPG bilayers and shows translocation of water molecules along the groove-like structure.

### Antimicrobial activity of wildtype LL-37 and mutants against *Staphylococcus aureus, Pseudomonas syringae, Streptococcus pyogenes* and *Escherichia coli*

The structure-based mutants of LL-37 were also used to estimate their activity against Gram-positive and -negative bacteria. As previously explained, we designed the E16A and R23A mutant peptides to investigate their implications in peptide interactions. We speculated that oligomerization was important for activity and mutants may reveal a different activity profile. Using the wildtype peptide and the bacterial strains *E. coli K12* (6.25 µg/ml; 1.4 μM), *S. aureus JE2* (25 µg/ml; 5.6 μM), *P. syringae* (6.25 µg/ml; 1.4 μM) and *S. pyogenes* (3.25 µg/ml; 0.73 μM) we found low minimal inhibitory concentration (MIC) values in the range of 3.25 to 25 µg/ml. Both mutants were essentially inactive against all four strains tested except R23A that maintained activity against *S. pyogenes* (6.25 µg/ml) (see Table SI). In summary, the structural analysis of LL-37 provided deep insights into the mode of oligomerization via interfaces which can be altered by mutation leading to altered activity.

An additive inhibitory effect of LL-37 was observed for *S. aureus* on plates containing daptomycin, an antibiotic used for severe methicillin-resistant *S. aureus* (MRSA) infections, and known to interact with bacterial lipid membranes^49^. Correlation of cell membrane lipid profiles with daptomycin resistance in methicillin-resistant *S. aureus* has been reported^49^. The presence of LL-37 in the medium increased the susceptibility of *S. aureus* against daptomycin significantly from 0.094 to 0.020 mg/ml (see Fig. S9). No additive inhibitory effects on *S. aureus* cultures containing the cell wall targeting component ceftobiprole or tigecycline antibiotics were found. Note that our strain from the CC8 complex suggests that this additive effect is not limited to strains showing USA600 genetic background^50^. In other Gram-positives such as *Enterococcus*^51^, the presence of a cell wall-targeting antibiotic increased bacterial susceptibility to LL-37 as well as that of daptomycin. These observations suggest direct interactions of the LL-37 molecules with charged components of the bacterial envelope in Gram-negative as well as in Gram-positive.

### Characterization of the LL-37 cell wall targeting mechanism in *E. coli* using cryo electron microscopy

**Figure 5:**
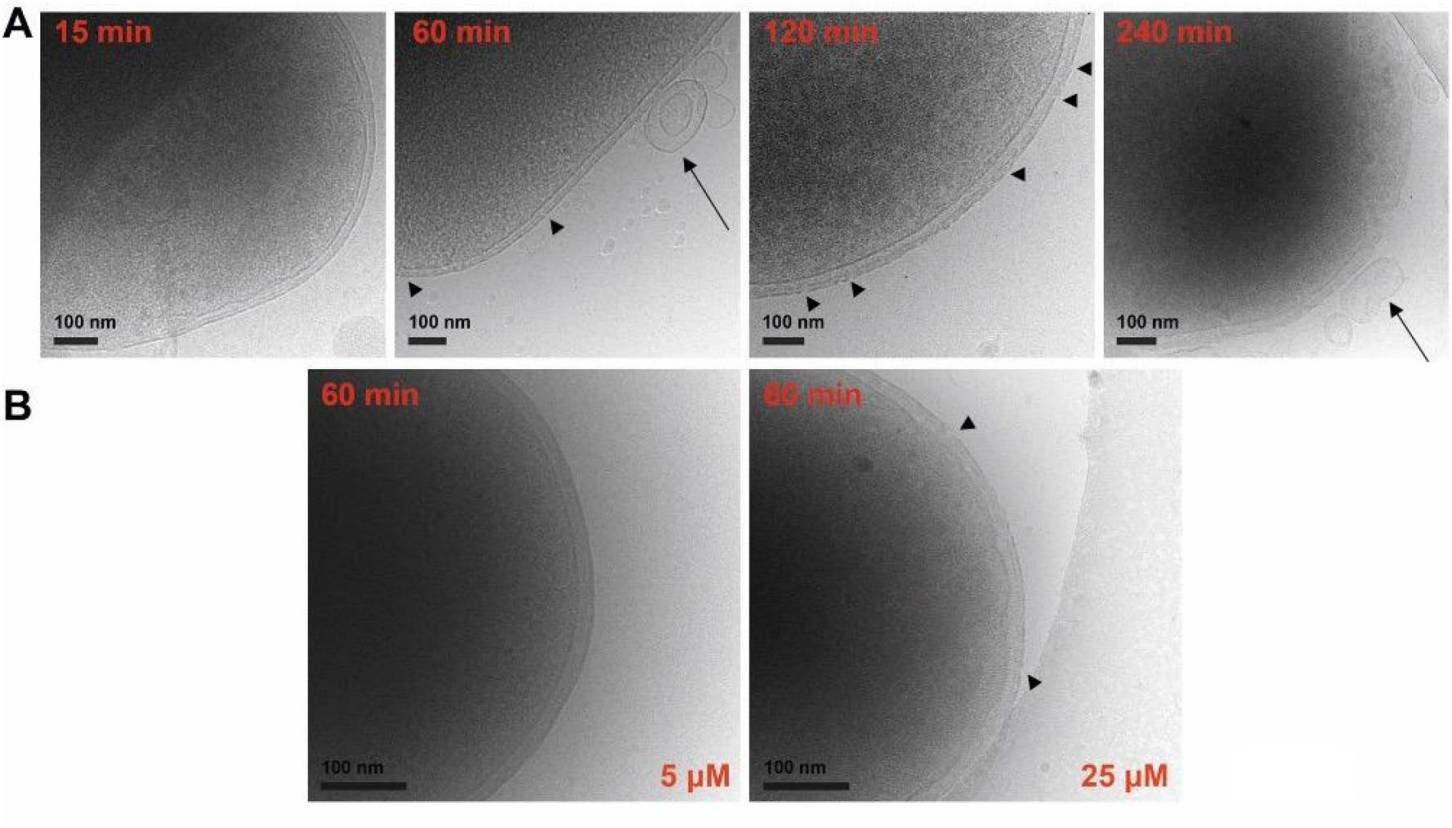
Cryo-EM images of *E. coli* in the presence of LL-37 show membrane disruption*.* **(A)** Electron micrographs of *E. coli* treated with LL-37 after 15, 60, 120 and 240 min. The images show the disruption of the *E. coli* membrane after incubation. In untreated cells (See Fig. S10A) the cell wall shows a distinct pattern of outer membrane, peptidoglycan and inner membrane. While the outer membrane was almost undisturbed after short time exposition, additional discontinuities (see black triangles) appear after longer incubation subsequently leading to the disruption of the inner membrane and the formation of extracellular stress vesicles (see arrows). **(B)** The effect of LL-37 is concentration dependent. At concentrations beyond or around the MIC (5 µM) the cells maintain their shape and the cell wall is undisturbed while at concentrations clearly above the MIC (25 µM) fragmentation patterns are rapidly visible.

In order to elucidate the mechanism of membrane disruption by LL-37 in vivo, *E. coli* cells were incubated with different peptide concentrations at different incubation times. First, we tested the cell wall alteration effects at 5 and 25 μM peptide concentrations (below/near the MIC and above MIC, respectively) for one hour and observed that significant changes were only visible at 25 μM (see Fig. 5B). We therefore used this concentration for the following experiments and incubated the cells for 15, 60, 120 and 240 min (see Fig. 5A). In *E. coli* control cells the micrographs show the three distinct and undisturbed constitutive layers of the cell wall: the outer membrane, peptidoglycan and inner membrane (see Fig. S10A). The time-dependent disruption process of the cell wall after incubation with LL-37 is demonstrated in Fig. 5A. While the outer membrane remains intact after 15 min of treatment, perforation becomes apparent after 60 min together with membrane blebbing and the secretion of stress vesicles. After 120 min a significant deformation of both membranes is apparent but no strong perforation of the inner membrane and the peptidoglycan can be recognized. Progressing cell death is further highlighted by changes in the contrast of the cytoplasmic space molecules after four hours and the formation of discontinuities in the inner and outer membrane, deformation of membranes and the peptidoglycan essentially disappeared. Images of cells incubated with LL-37 at 25 μM for 16 h show the presence of empty membrane envelopes after damage of both membranes and the entire release of cytoplasmic constituents (see Fig. S10B).

In order to study if the morphological changes in *E. coli* after LL-37 incubation are unique or share common features with related AMPs we tested Dermcidin and SMAP under the same conditions at the same concentrations. Dermcidin is unique in the negative net charge of -2 while SMAP has a strongly positive net charge of + 9, more similar to LL-37. *E.coli* cells were incubated for 1 h with 25 μM Dermcidin showing significant changes in the inner membrane while the outer membrane and the peptidoglycane remained essentially unchanged (see Fig. S11). For SMAP-29 we demonstrate that the outer membrane and the peptidoglycan are clearly visible after incubation of 1 h at 25 μM while the inner membrane shows deformations and changes in the morphology similar to Dermcidin (see Fig. S11). Collectively, these in vivo data of LL-37 indicate that the peptide perforates both membranes of *E. coli* cells in a time-dependent manner and this mechanism is clearly different to the mechanism employed by Dermcidin and SMAP.

## Discussion

**Figure 6:**
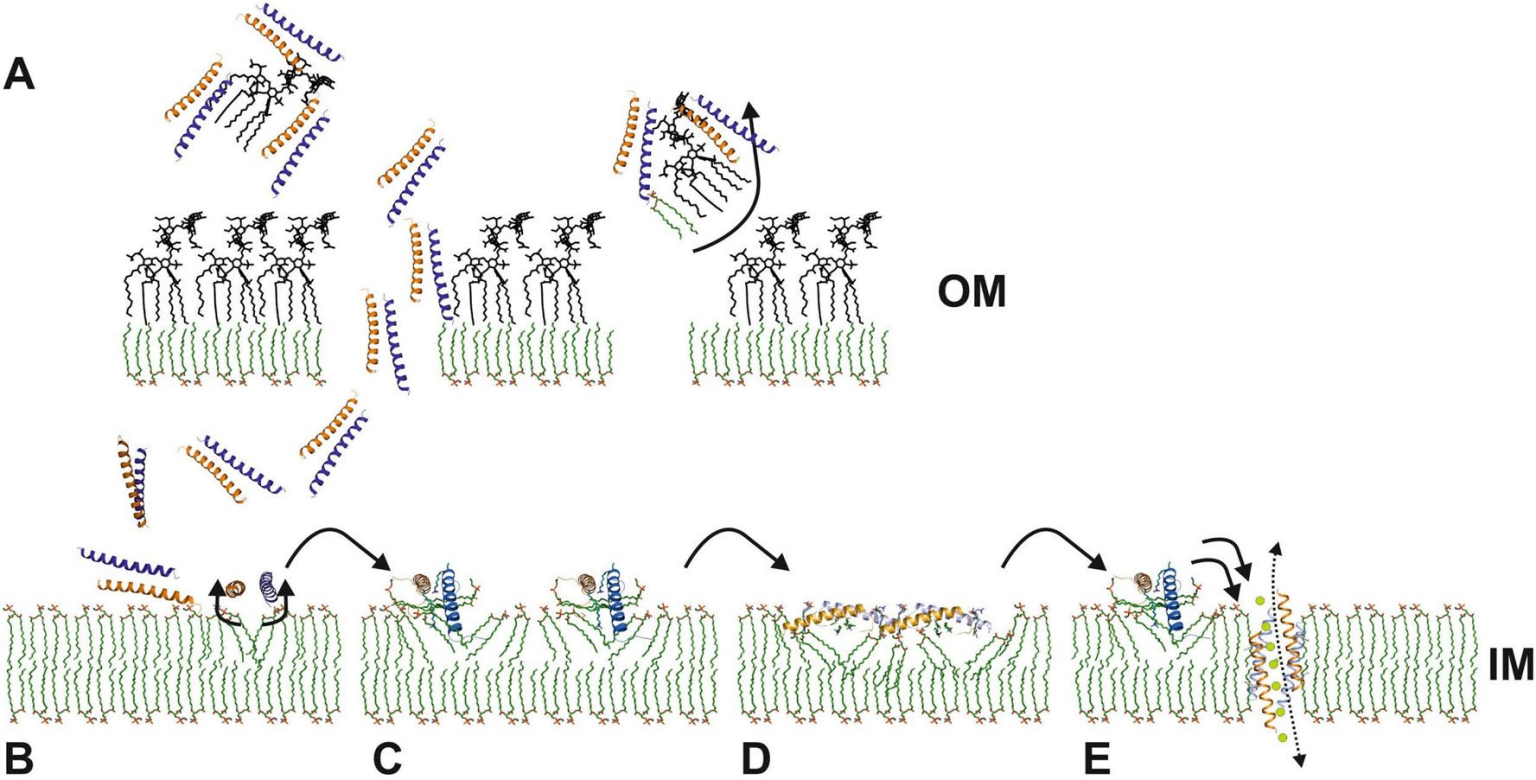
Proposed model of LL-37 interactions with the bacterial cell wall. A final killing step has been added to the previous proposed model of LL-37 interactions with the bacterial cell wall^24^. After initial electrostatic attraction with the outer membrane via LL-37/LPS or LL-37/LTA complexes **(A)**, LL-37 interacts with the PE of the inner membrane **(B)** and lipids may be extracted from the membrane **(C)** with the consequent formation of the activated peptide conformation LL-37 _LDAO-2_ **(D)**. **(E)** The final killing step of LL-37 follows by integration of the peptide into membranes as conducting channels, leading to the breakdown of the transmembrane potential.

Antimicrobial peptides are considered to be one class of molecules used as the basis for the development of novel antibiotics. In spite of their co-evolution with bacteria, they retained activity against many multidrug resistant bacterial strains^2^. One member of this class, the human cathelicidin LL-37 is probably the best studied antimicrobial peptide with a high activity against Gram-positive and -negative bacteria, including multi-resistant bacterial strains^10,52–54^. The success of LL-37 at killing is based on high physiological concentrations of the full length and a set of proteolytically truncated peptides and the ability to target various bacterial cell envelope molecules^55,56^. In a multistep mechanism, interactions with LPS, the peptidoglycan, the inner membrane and cytoplasmic proteins and DNA have been described, ultimately leading to cell death^56–60^. In contrast to the specific targets of traditional antibiotics, LL-37 interacts with essential cellular structures, disturbing their proper function and evading bacterial resistance mechanisms^12,34,61,62^. Understanding the targeting steps in structural detail would allow us to design LL-37 variants with increased efficiency for clinical applications. Therefore, we previously determined structures of LL-37 in complex with lipid-like molecules to understand the membrane-interacting mechanisms and showed the formation of peptide-detergent complexes as the basis for interactions with lipids followed by the formation of fibers (see Fig. 6)^24,35^. So far the structures of LL-37 showed monomers, dimers (in the presence and absence of detergents) and polymeric fibrils (shown by crystallography and electron microscopy). Fibril structures were formed only on the basis of LL-37/detergent- or LL-37/lipid-interactions followed by structural reorganization of termini and polymerization^24^. The formation of fibril structures are common features of various peptides with antimicrobial activity including phenol-soluble modulins (PSMs), BTD-2, the cationic peptide LAH4 and amyloid peptides (beta amyloid (Aβ) and IAPP)^25–28,63,64^. Peptides such as Aβ, IAPP or LL-37 are conformationally flexible and form lower oligomeric states which may translate into fibers due to external stimuli^23,26,65,66^. The mechanisms of fiber formation and their supramolecular organization depend on the peptide structure and plasticity. Oligomerization of amyloid peptides follows a pleiotropic spectrum of mechanisms but primarily includes β-fibrils of peptides in β-sheet conformation which assemble primarily via backbone interactions^67^. By contrast for the ɑ-helical PSMs and LL-37 peptides fibril formation is guided mainly through side chain interactions and can be induced by detergents and lipids in the case of LL-37^24,26^. The function of these fibrils for amyloids is largely unknown but it was speculated that their formation in LL-37 may locally increase their concentration as a step prior to membrane insertion and a protective “armor” function against bacterial attack was also proposed^23^. PSM fibrils are formed outside of cells and may serve to stabilize biofilms in *S. aureus* cultures^66^; however, more work is needed to find out if the fibrils discovered under in vitro conditions also form in the same way in vivo.

In this paper, we report complementary experiments to understand interactions between LL-37 and the cytoplasmic bacterial membrane in more detail. We used membrane-mimicking detergents and lipids and studied complexes with wildtype and LL-37 mutants using structural biology, biochemistry and molecular dynamics. Broad studies of this type in the field of antimicrobial peptides in general and LL-37 in particular are still rare but they are of great importance for the understanding of AMP mechanisms and their structure-based design.

First we used lipids and detergents to generate LL-37 complexes reflecting membrane-associated states and studied peptide-detergent crystals by crystallography. Initially, to optimize the concentration of detergent for crystallization we determined secondary structure changes using circular dichroism along with increasing DPC concentrations and selected a DPC concentration of 1% which clearly increased the content of ɑ-helix (see Fig. S4). Under these conditions we obtained crystals of an asymmetric tetrameric peptide channel formed by two identical dimers. We analyzed these dimers with respect to the previously published structures and found that the structure cannot be superimposed on LL-37 nor LL-37_DPC-2,_ respectively as dimers. However, the monomer structures can be superimposed with a lower r.m.s.d for the membrane-associated states (LL-37_DPC-4_/LL-37_DPC-2_ compared to LL-37_DPC-4_/LL-37) and both of these structures showed a partial disorder of the N-terminus in comparison to the detergent-free structure. LL-37 was also previously studied by NMR and here the peptide (in 1% SDS) shows a monomeric and bent structure which significantly deviates from the crystal structures^44^. The r.m.s.d. between the NMR structure and LL-37 is 2.6 Å (for 26 aligned CA positions), 2.4 Å to LL-37_DPC-2_ (27 aligned CA positions) and 2 Å to LL-37_DPC-4_ (26 CA positions aligned). In another NMR study a helix-break-helix model of LL-37 in 1% DPC was determined which is reminiscent of the LL-37_DPC-2_ conformation^45^. This NMR study also confirmed the flexibility of N- (residues 1–12) and C-terminus (33–37) in the presence of detergents and proposed a random coil structured termini rather than an α-helical structure. This agrees with the significant structural rearrangement visible in our X-ray structures of LL-37_DPC-4_ and LL-37_DPC-2_ after detergent exposure with DPC (see Figs. 1, S1, S2, S3). All crystallographic structures of LL-37 show two antiparallel helices, an arrangement which resembles the previously published crystal structures of DCD, magainin-2 and melittin (Mel)^35,68,69^ (see Fig. S12A). The primary interfaces are stabilized by hydrophobic residues and form elongated structures of 4 (LL-37_DPC-4_), 3.5 (Mag-2M) and 8 nm (DCD), a length sufficient to span biological membranes (see Fig. S12B). Oligomer interfaces at high resolution have been discovered only for the hexameric dermcidin channel and in this work, showing alternating hydrophobic (IF1) and hydrophilic interfaces (IF2 and IF3) in LL-37_DPC-4_ (see Fig. S12C). While the LL-37_DPC-2_ structure is indicative for the carpet-like model, the observation of a tetrameric channel structure in LL-37_DPC-4_ suggests the barrel stave model as another putatively final step of membrane targeting (see Fig. 6E)^35^. Formation of such LL-37 channels may originate at membranes after dimerization and oligomerization and the peptide oligomers may be moved into membranes in response to transmembrane potentials as shown for Dermcidin, latarcins and Magainin-2^35,70,71^. The targeting mechanisms are obviously complex and their description requires new models based on combinations of the enhanced scheme of Nguyen et al.^30^.

Could the tetrameric state of LL-37 resemble a possible conformation obtained in native membranes? We identified various structural features of LL-37_DPC-4_ underlining its potential to form similar channels in vivo: (I) the length of LL-37_DPC-4_ fits the biological membrane thickness (see Fig. 1, S1, S2); (II) the peptide interfaces show significant intermolecular interactions and follow an antiparallel orientation similar to the DCD channel, together with the alternating interfaces (see Figs. 1, S12); (III) the properties of the structure when integrated in membranes show minor unfavorable electrostatic violations with the membrane environment; (IV) two girdles formed by aromatic residues surround the periphery of the channel center at a distance of ~ 2 nm (see Fig. 1B). Such girdles, together with positively charged flanking residues, have frequently been observed in inner and outer membrane protein structures and appear to stabilize membrane proteins by the formation of protein-lipid contacts^72,73^.

To experimentally prove that the channel could also form under physiological conditions, additional experiments were conducted which demonstrate oligomerization as a prerequisite step towards channel formation: (A) based on cross-linking, size exclusion chromatography and analytical ultracentrifugation in the presence of detergents higher molecular oligomers could be demonstrated^24,74^. Cross-linking of LL-37 in the presence of detergents and lipids showed the formation of higher-molecular oligomers, although not only tetramers but also higher oligomeric states. In particular negatively charged lipids and detergents seem to interact more strongly with the peptide and also induce changes in the secondary structure (see Fig. 2); (B) according to the LL-37_DPC-4_ structure, a small conductivity was expected and planar lipid membrane measurements confirmed 13 pS in 200 mM KCl (65 pS in 1 M salt) which is in the range of the channel conductance in the range of the 81 pS (at 1 M KCl) for the hexameric DCD channel^35^; (C) finally, MD simulations in membranes proved channel structural stability within the time frame tested, and a similar tilting angle in membranes as previously determined for DCD was reached^35^. We are aware that the diameter of the channel, together with the MD results may imply that no ion permeation is possible. The channel is, e.g., clearly too narrow for the translocation of hexaquo-sodium or potassium ions; however it is also known for tetrameric ion channels (with a conductivity of 0.1–100 pS), that ions move along backbone carbonyl atoms through these narrow channels in the absence of a hydration shell and hence, it seems plausible that also LL-37 could act by a similar mechanism^75^ while the present simulation time might be too short to capture such events. (D) To test lipid affinities of LL-37 we used MD simulations of LL-37 in POPE/POPG (as prototypes for bacterial membranes) and POPC (mimicking mammalian membranes) membranes and verified those simulations experimentally by circular dichroism analysis. POPG and POPC have a zero spontaneous curvature but POPG has a negative charge while POPC is neutral at physiological pH^76^. POPE is formally uncharged but according to experimental data in nanodiscs is negatively charged at pH 7.4^77^. In our MD simulations of the LL-37 tetramer the number of hydrogen bonds with POPE/POPG lipid membranes was clearly greater than with a POPC. This difference in affinity was also observed in earlier MD simulation using LL-37 as a monomeric peptide showing a higher stability in POPG rather than POPC^78^. To experimentally verify this calculated stability difference of LL-37 (from MD) in the same lipid systems used for MD we applied LL-37 to mixtures of PE/PG and PC at different ratios. While the folding propensity along with the increase in secondary structure was higher for the negatively charged PE/PG vesicles in particular for ratios of peptide/lipid (1:10—see Fig. 2) the presence of the uncharged PC vesicles at all ratios decreased the intensity of the a-helical signal. The differential binding of LL-37 to lipids of different charges (PG/PE relative to PC) has previously also been shown by Sood et al. and highlights the biologically important function of LL-37 to discriminate target versus host cell membranes^79^. Previously we have used the DPC detergent carrying the same head group as PC for crystallization and circular dichroism. Interestingly, in contrast to PC the spectra showed a clear increase in secondary structure consequently folding propensity upon increased detergent concentrations. This effect could possibly be due to the physical structure of a micelle relative to a vesicle in diameter and shape (detergent micelle strongly bent relative to a vesicle)^79^. Taken all data together, we can provide biochemical data (raised under points A–D) which are in support of the LL-37 assembling into the LL-37_DPC-4_-like structure tested under different conditions (lipid membranes, pH, salt) thereby suggesting that the channel formation under ‘crystallization conditions’ may also occur in artificial or biological membranes.

Interactions between antimicrobial peptides and the bacterial cell envelope at higher resolution using cryoelectron microscopy are rare. When we started our investigation we imagined that the peptide would cross the outer membrane via outer membrane channels and used mutants of the Keio collection to test this hypothesis^80^. We used 20 knockout mutants of porins and proteins of the Ton and Tol complex but we did not obtain evident results. Next we tested the interactions of LL-37 with the bacterial cells in an in vivo setup. Using high resolution cryoelectron microscopy, images were recorded, clearly showing the three different layers of the cell wall; the outer membrane, the peptidoglycan and the inner membrane. Based on the resolution acquired for wildtype cells we were hoping to be able to specifically investigate the individual influence of LL-37 on these substructures and to link the activity to interactions studies with LPS, LTA and lipids. Wildtype *E. coli* cells were treated with LL-37 and the peptides SMAP-29 and DCD to compare if similar morphological changes would appear. The effects of LL-37 on the cell wall are time and concentration dependent, and clear discontinuities in the outer membrane develop after 120 min and cells start to become leaky with clear gaps in both membranes allowing for the release of cytosolic compounds. In contrast, the mechanism by which DCD acts on the same cell type yields different morphological changes (see Fig. S11). DCD is a unique peptide due to its charge properties (surplus of negative charges) and should only weakly interact with the negatively charged LPS or LTA molecules, respectively^81^. This unique property is reflected by the fact that the outer membrane remains intact while the inner membrane shows strong deformation and breaks. SMAP-29, although even more strongly positively charged than LL-37, shows a similar influence on the bacterial cell wall to DCD, acting primarily on the inner membrane but leaving the outer membrane and the peptidoglycan layer largely intact. Membrane deformations also occur here at a very early time points, however the outer membrane remains intact while the inner membrane collapses and fragmentation is visible. In other experiments where *E. coli* cells were exposed to LL-37 and other cathelicidins the authors found stress vesicles also observed in our studies and wrinkles after an earlier time point of incubation^82,83^. These observations were concentration dependent but occurred already at concentrations below the MIC and strong damage of the cell was observed at concentrations above the MIC and the overall killing time of *E. coli* was faster typically within minutes^82^.

One of the major aims of this work was also to find a better model describing interactions between the antimicrobial peptide LL-37 and bacterial cells focusing on the bacterial cell wall. Based on previous and unpublished studies we propose a mechanistic model which highlights LL-37 interactions with LPS and LTA as the first step of targeting. These interactions at the bacterial surface lead to the extraction of LPS to form holes in the outer membrane. Higher oligomerization state and fiber formation may be the next step induced by interactions with the cytoplasmic membrane. These fibers may be necessary to increase the local peptide concentration and to locally extract lipids from the cytoplasmic membrane after peptide interactions. A transmembrane potential may be necessary to pull the channel into the membrane (e.g. in case of DCD). The channel formation leads to the breakdown of the transmembrane potential and ultimately cell death (see Fig. 6). As the formation of the tetrameric pore would require a biological membrane together with ion transmembrane gradients, we assume that this channel would be stable only in the context of the inner membrane. Therefore, we cannot postulate the tetramer as a general oligomeric form of LL-37 in every context or lipidic membranes rather as one of several possible snapshots of LL-37 structures. In summary: In this and a previous paper on LL-37 we showed significant structural variability exhibited by LL-37 and we suspect that they are only a subset of other physiologically important conformations. This structural flexibility might allow LL-37 to adapt and interact with LPS and and also the outer or inner membranes, respectively, which is a prerequisite for the various activities of the peptide.

## Summary

This work shows new peptide-peptide and peptide-detergent interaction sites of LL-37 deciphered at atomic resolution, together with various modes of oligomerization. We show for the first time the unusual structural plasticity of this peptide towards several cellular target molecules shown by four crystal structures. Structural work leading to the identification of residue specific interactions in these complexes can be used for the design of novel LL-37 peptides with e.g. increased stability of dimer interfaces, hence improved activity. Interactions of the four different structures are assembled in Fig. S3 and residues primarily involved in these interactions are located in the peptide center from Lys12 to Phe27. So far, point mutants E16A and R23A and their altered activity indicate the correctness of the structures and the potential to change the activity based on single mutations, also in the context of cellular targets. Terminal deletion mutants LL-27 and FR-22 were inactive in killing bacteria but could be activated by adding detergents which opens another venue for the development of new peptide antibiotics based on structural information. These detergents seem to stabilize certain portions of the dimer interface and enhance the ability for oligomerization and enhance the antimicrobial activity. The different peptide-peptide and peptide-detergent interactions are displayed in Fig. S13, and they indicate that the central part of the sequence is the most important for structure maintenance. Deletion mutants created based on this information, and truncated peptides from the literature, prove the importance of these residues^24^. However, important structural information regarding the complexes between LL-37 and LTA or LPS is still missing and they would add on another important piece of knowledge. Future studies can build on this information to create smaller LL-37 variants with similar activity which may be further introduced in preclinical and clinical studies.

## Materials and methods

### Peptides, lipids and detergents

All peptides used in our study were chemically synthesized and purchased from Peptide2.0 (www.peptide20.com) at purities higher than 95%. DPC was purchased from Affymetrix, while cholesterol and 3-sn-phosphatidylethanolamine (PE) were purchased from Sigma-Aldrich. 1,2-diphytanoyl-sn-glycero-3-phosphocholine (DPhPC) and phosphatidylglycerol (PG), dissolved in chloroform were purchased from Avanti Polar Lipids. Phosphatidylcholine (PC) from soybeans was acquired from the List of European Pharmacopoeia Reference Standards (EDQM).

### Crystallization and crystallographic studies of LL-37

Crystallization of the tetrameric channel was conducted with peptide-detergent mixtures (20 mg/ml LL-37, 0.5% DPC) in a 2 mM sodium phosphate pH 6.8 buffer, using the commercial screens from Jena Bioscience, Qiagen and Molecular Dimensions. Sitting drops of 400 + 400 nl (peptide + reservoir) were prepared by a Mosquito robot (TTP Labtech) and the progress of crystal formation was monitored using the Formulatrix Rock imaging system. Crystals were mounted from crystallization drops and data were collected at the synchrotron source SLS (Swiss Light Source, Villigen, Switzerland—beamline PX10). Data were recorded on the Pilatus detector 6 M (Dectris) at 100 K with beam attenuation of 20–50% at beamline PX10. Data were processed using the XDS/XSCALE program package^84^. The high resolution structure of monomeric LL-37 crystallized in DPC^24^ was employed as a search model to solve the tetrameric structure obtained in the presence of DPC. The structure was refined by Refmac^85^, Phenix^86^ and BUSTER^87^ and manually modelled using the COOT program package^88^. Based on F_O_-F_C_ difference maps, detergent molecules were added and refined together with the peptide model. The geometry of the structure was validated using the Molprobity server (https://molprobity.biochem.duke.edu/). All refinement and model statistics are given in table SII.

### Vesicle preparation

The phospholipid vesicles were prepared by pipetting the lipids (chloroform resuspended) in borosilicate-glass culture tubes with teflon-lined screw caps. The organic solvent was removed by evaporation under a nitrogen stream by using a dry heated nitrogen evaporator. The dried lipids were hydrated by adding 5 mM phosphate buffer pH7, 0.1 M NaCl with the consequent obtaining of multilamellar vesicles (MLV). Small unilamellar vesicles (SUV) were obtained by vortexing and sonication in the bath sonicator for 15 min at room temperature^89^. After sonication, the peptide was added to the SUVs at the indicated ratio and incubated at room temperature for 5 min before CD and cross-linking performance.

### Characterization of peptides by circular dichroism (CD)

40 μM (CD with PE:PG or PC) in 100 mM NaCl, 5 mM phosphate, pH 7, was used for the secondary structure determination using a CD JASCO J-815 spectrophotometer (Jasco Spectroscopic Co. Ltd., Hachioji City, Japan). Peptides were analyzed at 20 °C and a scanning speed of 100 nm/min with a band width of 1 nm. All spectra were recorded at 0.5 nm resolution and data were reported as differences in molar absorption. The samples were measured in quartz precision cuvettes with a path length of 1 mm. DPC was added at 0.14 mM–5.7 mM concentrations and PE:PG (3:1) and PC at 1:0.1–1:10 (LL-37:lipid ratio).

### Cross-linking experiments

LL-37 at a concentration of 40 μM (with lipids) was subjected to cross-linking using the amino-reactive cross-linker BS3 (Thermo Scientific, Pierce Biotechnology, Inc.). BS3 at a final concentration of 1 mM was added to the samples used previously for the CD and the incubation was continued for 1 h at 37 °C while shaking. The reaction was stopped by addition of 50 mM Tris–HCl, pH 8 and the mixture was kept for 15 min at room temperature. The sample was mixed with a denaturing Laemmli buffer and boiled for 10 min at 95 degrees. Samples were loaded on Novex 10–20% tricine gels (Invitrogen Life Technologies) and analyzed after silver staining for the cross-linking done in the presence of lipids.

### Electrophysiology measurements

Planar lipid bilayers were obtained from GUVs prepared by using electroformation method in an indium tin oxide (ITO) coated glass chamber connected to the Nanion Vesicle Prep Pro setup (Nanion Technologies GmbH, Munich, Germany)^90^. Lipid-containing solution, 10 mM of 1,2-diphytanoyl-sn-glycero-3-phosphocholine (DPhPC) with 10% cholesterol, dissolved in chloroform, was deposited on the ITO-coated glass surface. After total evaporation of the solvent the lipids were assembled in a perfectly dehydrated lamellate phase. 300 μl of a non-ionic intracellular solution, sorbitol (1 M) was added to the dry lipid film. The process of electroformation was controlled by the Vesicle Prep Pro instrument and all parameters (amplitude, frequency, duration, etc.) for the electroformation were programmed in the VesicleControl software (Nanion Technologies GmbH, Munich, Germany). Generally, an alternating voltage of 3 V peak to peak was applied with a progressive increase for the rise time and a decrease for the fall time to avoid abrupt changes, which otherwise might rupture the formed GUVs. The frequency of the alternating current was 5 Hz and was applied to the ITO-slides over a period of 2 h at room temperature. After successful swelling, the vesicles were used directly for the planar lipid bilayers formation. For the formation of the planar lipid bilayer, the GUVs were positioned onto the aperture of the Port-a-Patch automated patch clamp system (Nanion Technologies GmbH, Munich, Germany), using borosilicate glass chips with an aperture diameter of approximately 1 μm. Typically, (−)10 to (−)40 mbars were sufficient for reliable positioning within a few seconds after GUV addition. When the GUVs touch the glass surface of the chip, they burst and form planar bilayers^91^. LL-37 was reconstituted by simple addition to the top of the chip where the bilayers were sitting. After a period of incubation we started to see activity that mainly conducted to the breaking of the membrane when used at the purification concentration directly. After several dilutions, single channel activity could be observed. The recordings were done at a transmembrane potential of + 100 mV with 200 mM KCl, 10 mM HEPES at pH 7 inside and pH 4 outside.

### Molecular dynamics simulations

All molecular dynamics (MD) simulations were performed using the CHARMM36 force field^92,93^ and the GROMACS 4.6.5 software^94^. Short-range pairwise electrostatic interactions were considered up to 1.2 nm and beyond it calculated with the particle-mesh Ewald method^95^. Furthermore, van der Waals (VdW) interactions were considered up to a distance of 1 nm which smoothly turn off at 1.2 nm. As a starting structure for the MD simulation setup, the crystal structure of the LL-37 tetramer was inserted into a heterogenous bilayer built of 1-palmitoyl-2-oleoyl-sn-glycero-3-phosphatidylethanolamine and 1-palmitoyl-2-oleoyl-sn-glycero-3-phospo-(1′-rac-glycerol) (POPE and POPG) lipids at a ratio of 3:1 generated using CHARMM-GUI Membrane Builder^96,97^. This negatively charged bilayer composed of POPE and POPG lipids should yield a reasonable model for bacterial inner membranes^98^. The bilayer was aligned parallel to the xy plane and centered in the z direction. In a subsequent step, the lipid bilayer together with the LL-37 channel was solvated in a periodic TIP3P water box with 1 M KCl resulting in a system size of approximately 80 Å × 80 Å × 100 Å (see Fig. 4A). A similar procedure was used for setting and equilibrating the homogenous POPC, POPE and POPG bilayer systems with LL37 embedded in the respective membrane and a 1 M KCl solution. All four systems were equilibrated using the well-established protocol developed in the CHARMM-GUI Membrane Builder and followed by two 200 ns production runs for each setup in a constant temperature and pressure (NPT) ensemble achieved by a Nóse-Hoover thermostat^99,100^ with a coupling constant of 1 ps and a semi-isotropic Parrinello-Rahman barostat^101^ at 1 bar with a coupling constant of 5.0 ps. An integration time step of 2 fs was used by constraining the hydrogen bonded atoms to their equilibrium lengths with LINCS algorithm^102^. The tilt angle is defined as the cosine of the angle between two vectors, i.e., principal axis of the starting structure and the respective frames in the trajectory. Hydrogen bonds were calculated using the g_hbond tool based on the geometric criteria for the distance between donor–acceptor being less than or equal to 0.35 nm and the angle between hydrogen-donor–acceptor being less than or equal to 30°.

### Microbial assays of LL-37 peptide

Potential synergistic effects of LL-37 and antimicrobial agents were evaluated on a solid medium (Muller Hinton agar plate). Etest strips (bioMérieux, La Balme, France) with antibiotics from three distinct families targeting cell wall components (ceftobiprole, tigecycline) or lipid metabolisms (daptomycin), were performed on Muller Hinton agar plates containing LL-37 at a concentration of 25 mg/l compared to the same medium free of LL-37. Solid medium (20 ml) was kept at 45 degrees and enriched with LL-37 at indicated concentration under constant supervision. Plates were then incubated at ambient temperature until solidification. The inoculum suspension was prepared from overnight cultures at a standardized titer of 0.5 McFarland and inoculated with a cotton swab over the entire surface of the Mueller–Hinton agar plate by swabbing in three directions. Etest containing indicated antibiotics were added onto the agar surface before incubation for 16 h at 37 °C. Three independent replicates were performed and showed the same result.

### Preparation of bacterial cells treated with AMPs for cryo-EM and data collection

*Escherichia coli* K12 cells were grown at 37 °C at 200 rpm to an OD_600_ of 0.6. LL-37, SMAP-29 and Dermcidin were added to 1 ml cultures to reach a final concentration of 5 µM (23 µg/ml) or 25 µM (115 µg/ml), depending on the experiments, and cultures were further incubated at 37 °C while shaking at 300 rpm (Eppendorf Thermomixer, Hamburg, Germany). After increasing times (15, 60, 120, 240 min and overnight) 100 μl of the samples or controls without AMPs were taken and cells were pelleted at 3000*g for 10 min at 4 °C. The cell pellets were resuspended using 20 μl of culture solution in order to concentrate the sample and reach a sufficiently high cell density for the visualization in the microscope.

To prepare vitrified grids for cryo-EM experiments, 4 µl of the concentrated sample solution in the case of the experiments with *E. coli* cells or from the vesicles incubated with LL-37 or LL-37 labeled with nanogold were applied onto glow-discharged Quantifoil R 2/1 200-mesh holey-carbon grids. Later the grids were blotted with a paper filter and were abruptly plunged in a liquid ethane bath, cooled with liquid nitrogen to − 196 °C, using a Vitrobot (FEI). Vitrified grids were cryo-transferred into a 626 DH cryo transfer holder (Gatan Inc.) and manually analyzed on a JEM-2200FS/CR (JEOL, Ltd.) transmission electron microscope equipped with a field emission gun (FEG) operated at 200 kV. No-tilted zero-loss two-dimensional (2D) images were collected under low-dose conditions, with a total dose of the order of 10–20 electrons/Å^2^ per exposure, on a 4 K × 4 K 15 µm pixel Ultrascan4000™ CCD camera (Gatan Inc.), at defocus values ranging from 2.0 to 4 µm. The in-column Omega energy filter of the microscope helped to record images with improved signal-to-noise (SNR) ratio by zero-loss filtering, using an energy slit width of 15 eV centered at the zero-loss peak of the energy spectra. Digital images were recorded using DigitalMicrograph™ (Gatan Inc.) software at different nominal magnifications, between 30,000 × and 60,000 ×, resulting in a final pixel size between 3.6 Å/pixel and 1.7 Å/pixel respectively.

## Supplementary information

Supplementary Information.

## Data Availability

The structure of LL-37_DPC-4_ has been deposited at the PDB database under the following number: 5G1J.
