# Supplementary material for: The structure of the antimicrobial human cathelicidin LL-37 shows oligomerization and channel formation in the presence of membrane mimics
Source: Sci Rep. 2020 Oct 15;10:17356. doi: 10.1038/s41598-020-74401-5 (PMC7562864; doi:10.1038/s41598-020-74401-5)
Supplement: Supplementary file 1 — Supplementary Information. [file 41598_2020_74401_MOESM1_ESM.docx]

**The Structure of the Antimicrobial Human Cathelicidin LL-37 Shows Oligomerization And Channel Formation In the Presence of Membrane Mimics** by Enea Sancho-Vaello, David Gil-Carton, Patrice François, Eve-Julie Bonetti, Mohamed Kreir, Karunakar Reddy Pothula, Ulrich Kleinekathöfer, Kornelius Zeth

**Supplementary figures:**


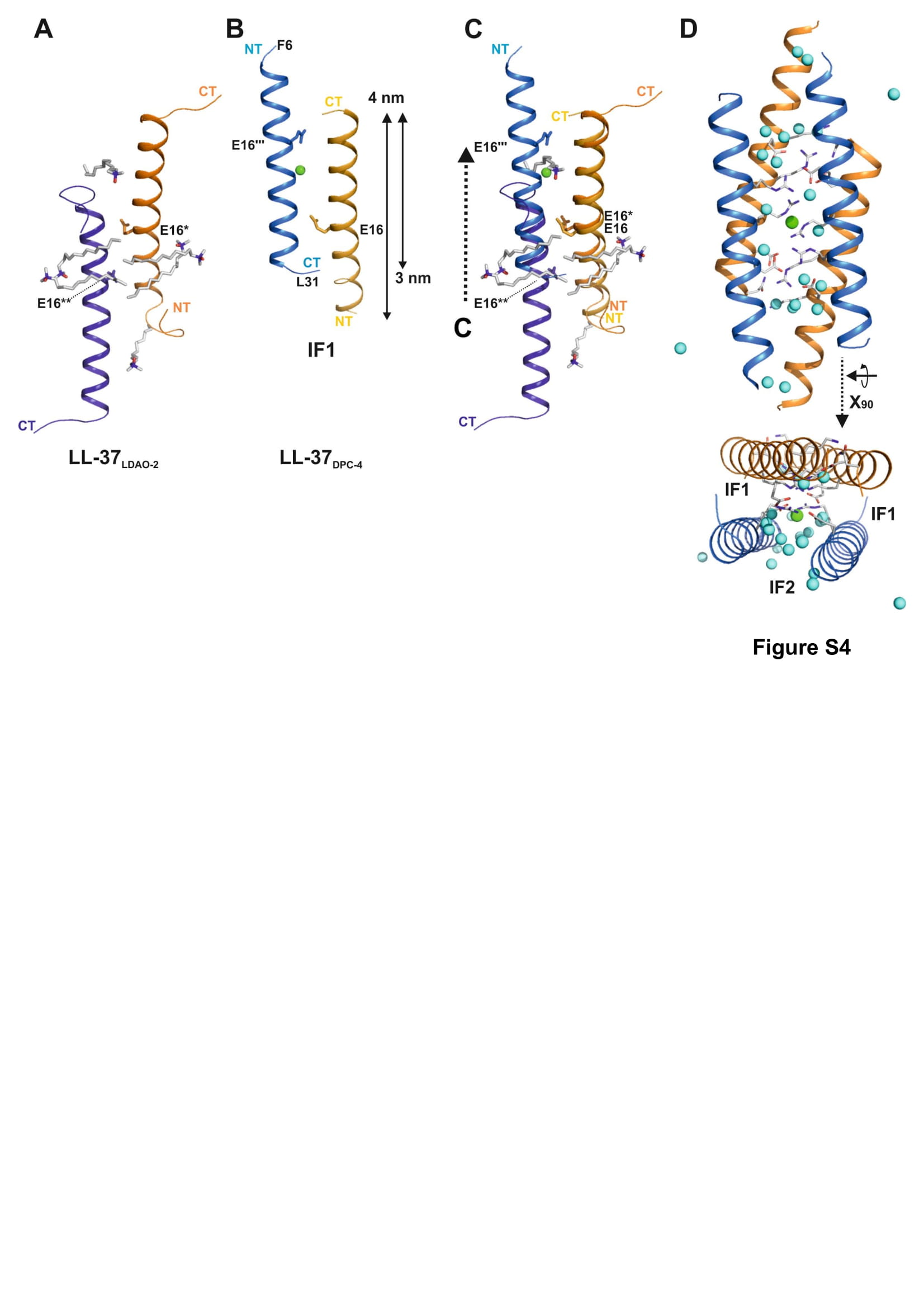


**Figure S1: –**  ***Comparison of LL-37 _LDAO-2_ and LL-37 _DPC-4_ dimer structures and water distribution in the putative channel.*** (A) Structural representation of LL-37 _LDAO-2_ with the two monomers color coded in orange and blue. The central residue Glu16 is marked and used to monitor the relative structural changes between LL-37 _LDAO-2_ (PDB 5NNK) and LL-37 _DPC-4_. (B) Structure of the IF1 dimer in LL-37 _DPC-4_ highlighting the same Glu16 residue. (C) Superposition of the structures according to the residue sequence using the monomer highlighted in orange. The second monomer of LL-37 _DPC-4_ is shifted along the second helix axis by about four turns. (D) Top and side view of LL-37 _DPC-4_ and distribution of water molecules marked in cyan is displayed at the putative pore entrances sites and along the IF2 interface.

**
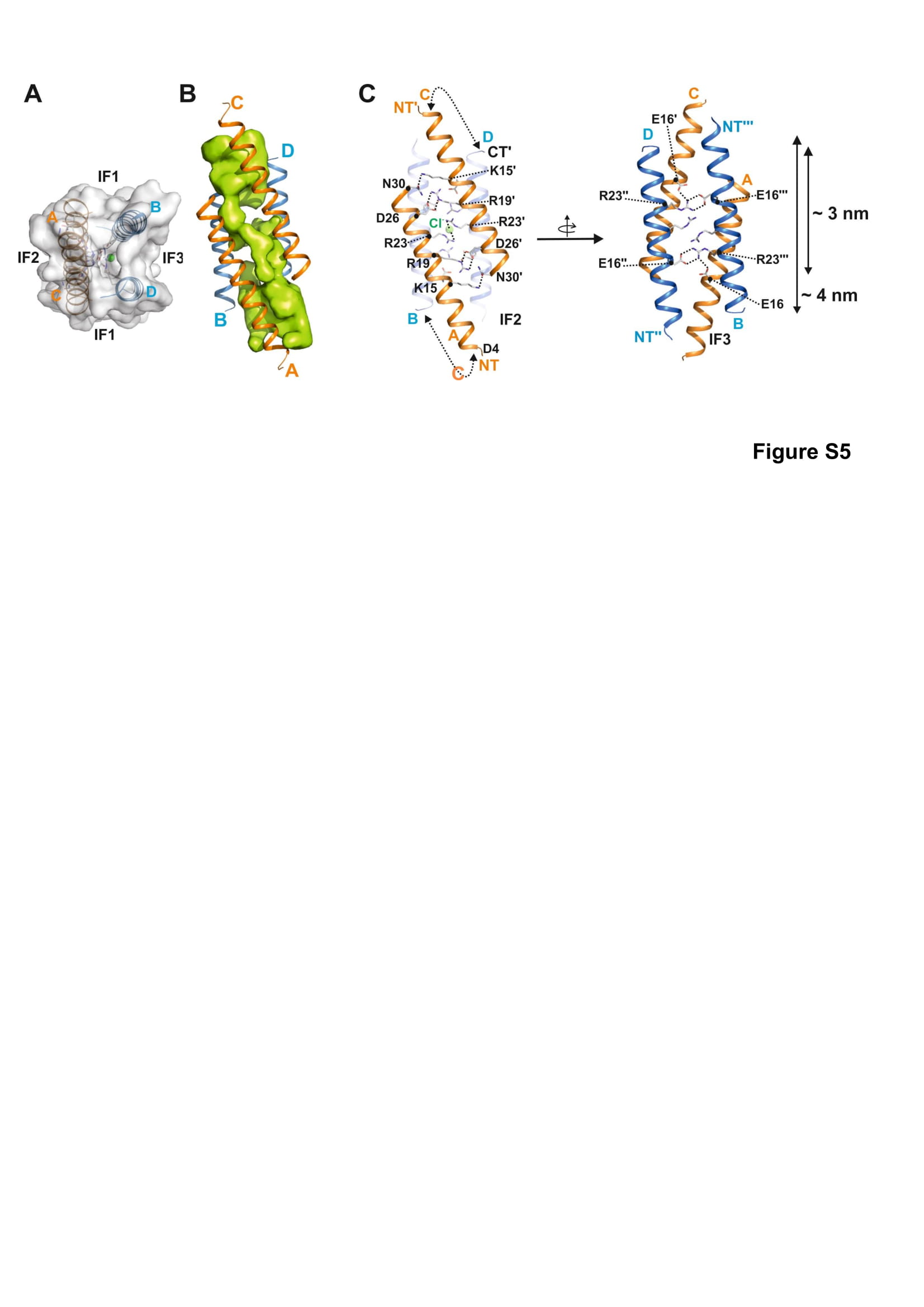
**

**Figure S2: –**  ***The structure of LL-37_DPC-4_ provides insights into the putative transmembrane channel architecture.*** (A) Top view onto the cartoon representation of the tetrameric channel embedded in a surface envelope. Helix pairs A/B and C/D are structurally identical and their interfaces IF1 are equivalent while, due to their asymmetric arrangement along the channel axis interfaces IF2 and IF3 are different. (B) Ribbon model of LL-37 with the accessible volume represented by green envelopes demonstrating a constriction site at the centre of the pore. (C) The channel is shown from the membrane perspective and the dense network of salt bridges and H-bonds inside the channel is indicated.


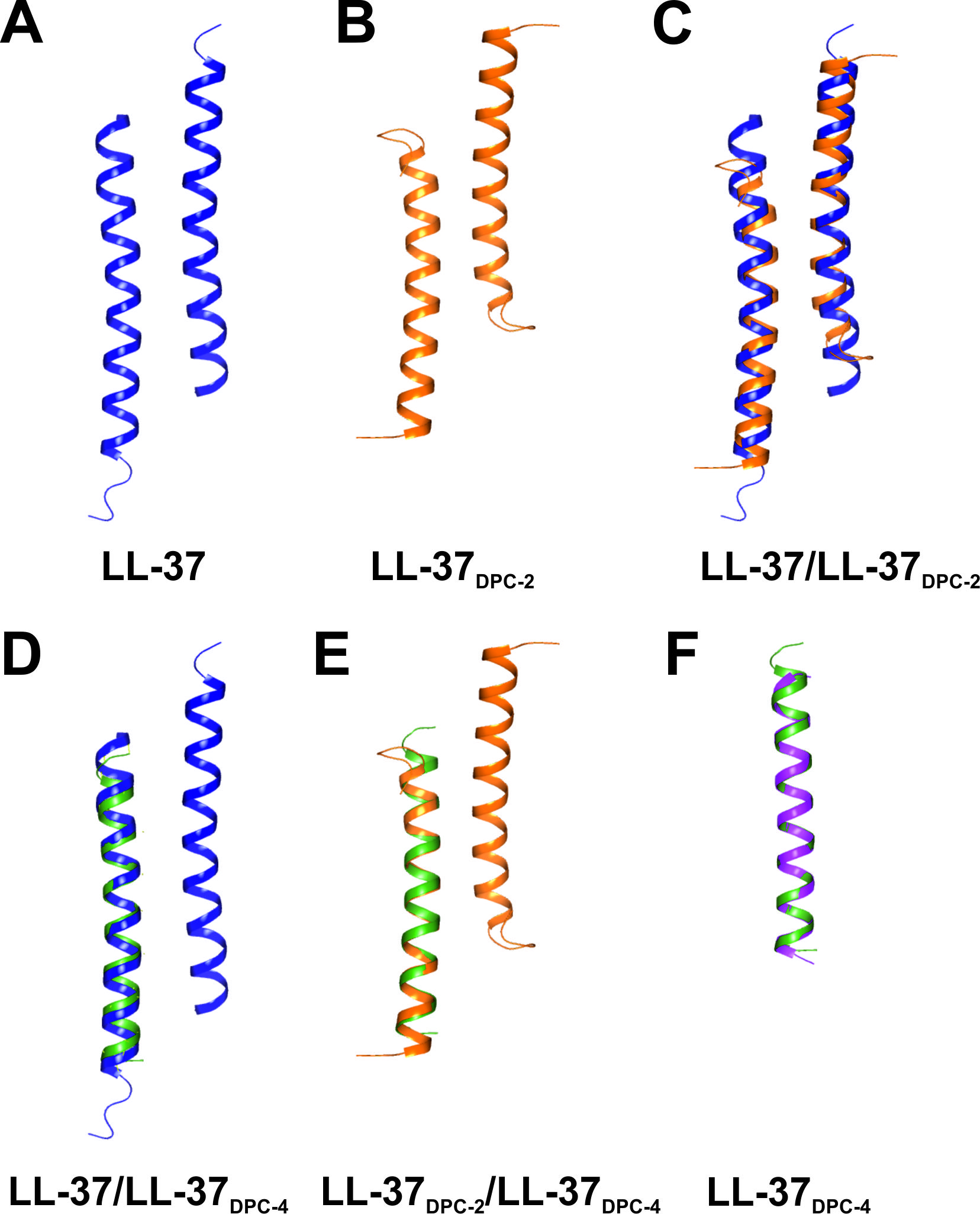


**Figure S3: –**  ***Comparison of crystallographically determined LL-37 structures.*** (A) LL-37 dimer in the absence of detergent, called LL-37 (PDB 5NNM); (B) LL-37 dimer in the presence of DPC, called LL-37_DPC-2_ (PDB 5NNT); (C) structural superposition of LL-37 and LL-37_DPC-2_ ; (D) structural superposition of LL-37 and a monomer of LL-37_DPC-4_ (PDB 5G1J, this paper) shown in blue and green, respectively; (E) structural superposition of LL-37_DPC-2_ and a monomer of LL-37_DPC-4_ shown in orange and green, respectively; (F) structural superposition of two monomers in LL-37_DPC-4_ asymmetric unit. They need to be shifted relative to each other by five turns to form the LL-37_DPC-2_ structure.

**
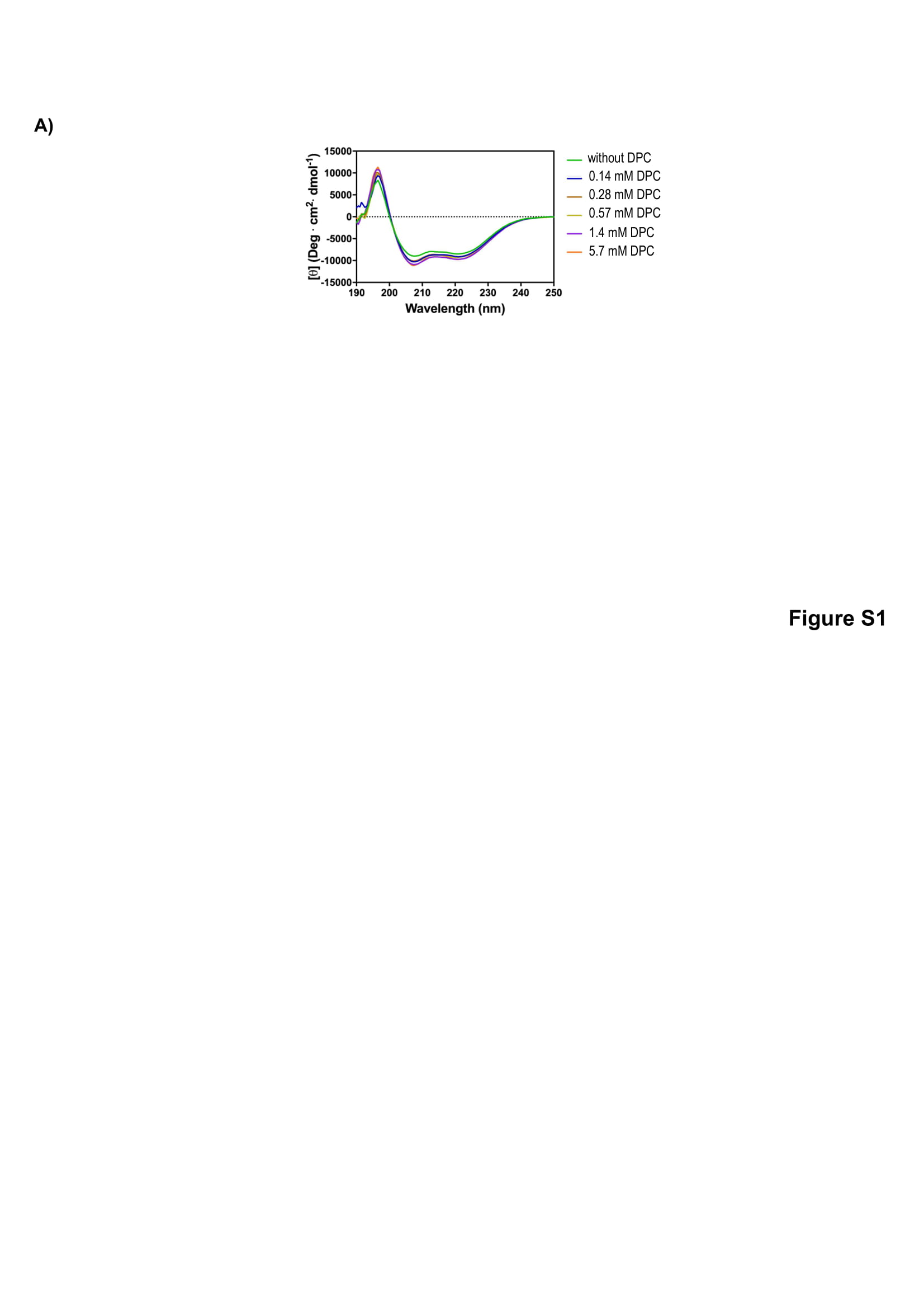
**

**Figure S4: *CD spectra of LL-37 in the presence of increasing amounts of DPC.*** Increasing concentrations of DPC (0.14 mM blue line, 0.28 mM brown line, 0.57 mM ochre line, 1.4 mM purple line and 5.7 mM orange line) were added to a solution of 0.1 mM LL-37 (green line) and the folding process was monitored. LL-37 is a well folded peptide in the presence of 100 mM NaCl and 5 mM phosphate buffer so that the detergent DPC does only marginally change the helical content.

**A B**


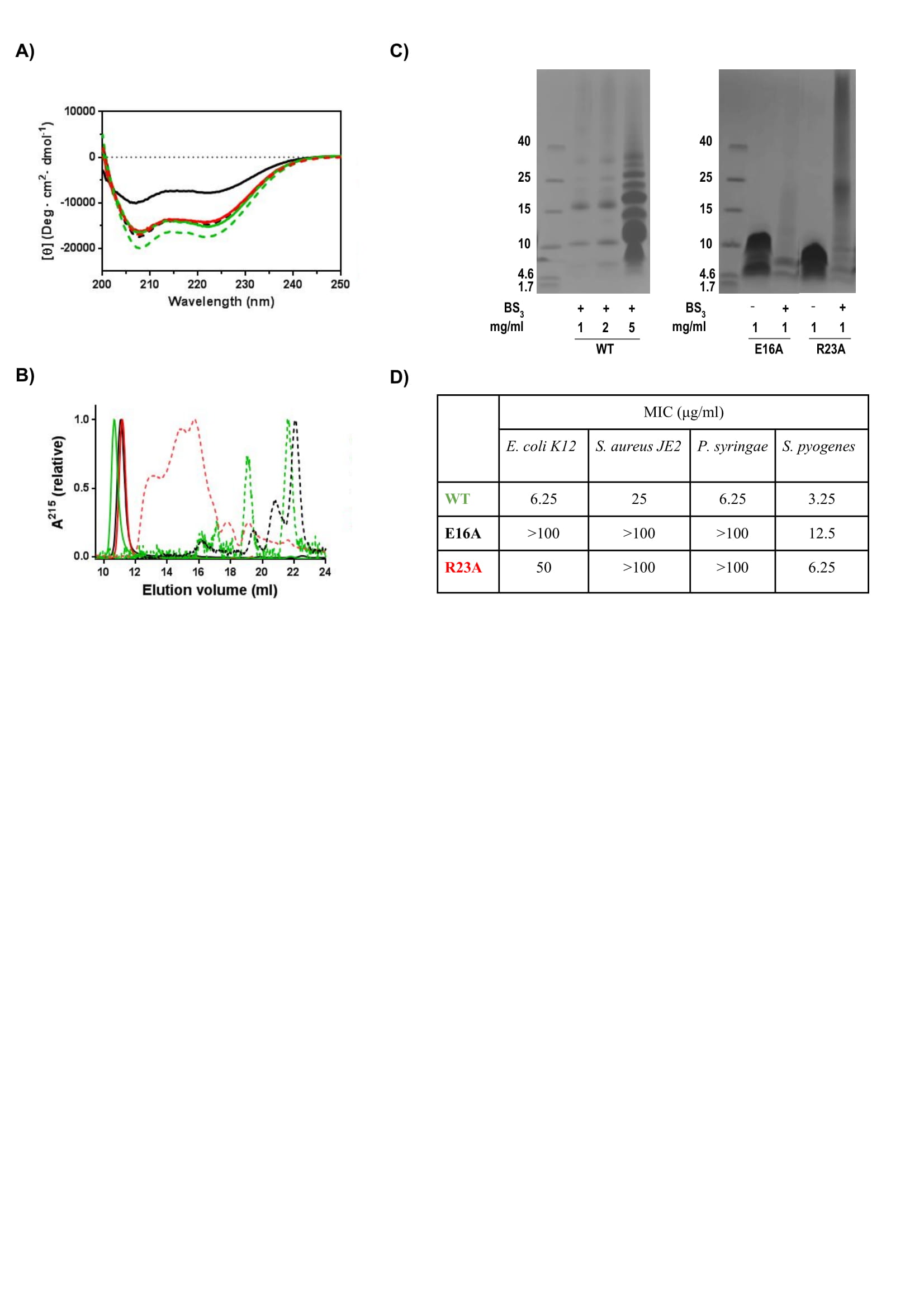

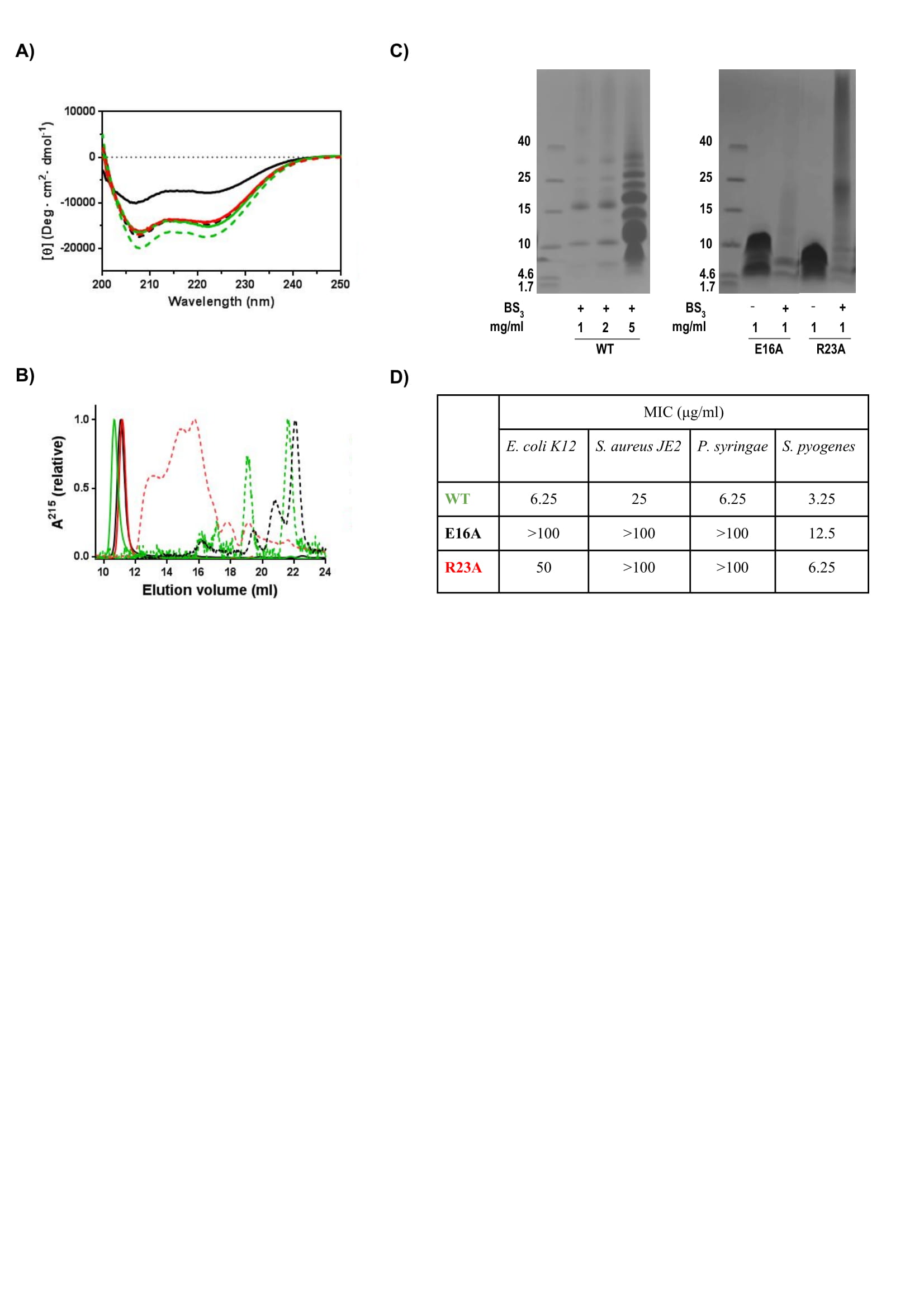


**Figure S5: – *Biochemical analysis of E16A and R23A LL-37 mutants.* (A)** Circular dichroism of WT, E16A and R23A in the absence (solid line) and presence of 0.2% DPC (dashed lines). Wild-type LL-37, E16A and R23A are shown in green, black and red, respectively. **(B)** Cross-linking of WT (left) and E16A and R23A (right) using BS3 as crosslinker (cropped gels; full-length gels are presented in Figure S14). The patterns exhibited by E16A and R23A are completely different to the one exhibited by wild-type.


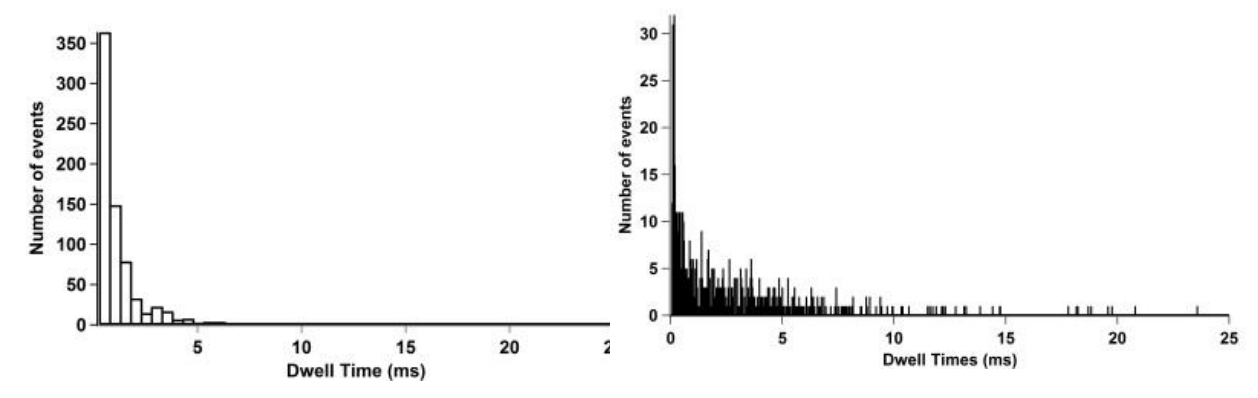


**Figure S6: – *Dwell time of the closed state (left) and the open state (right) of the LL-37 channel.*** The dwell time of the closed time was 0.7 ms while the open state had a longer opening of 1.8 ms.


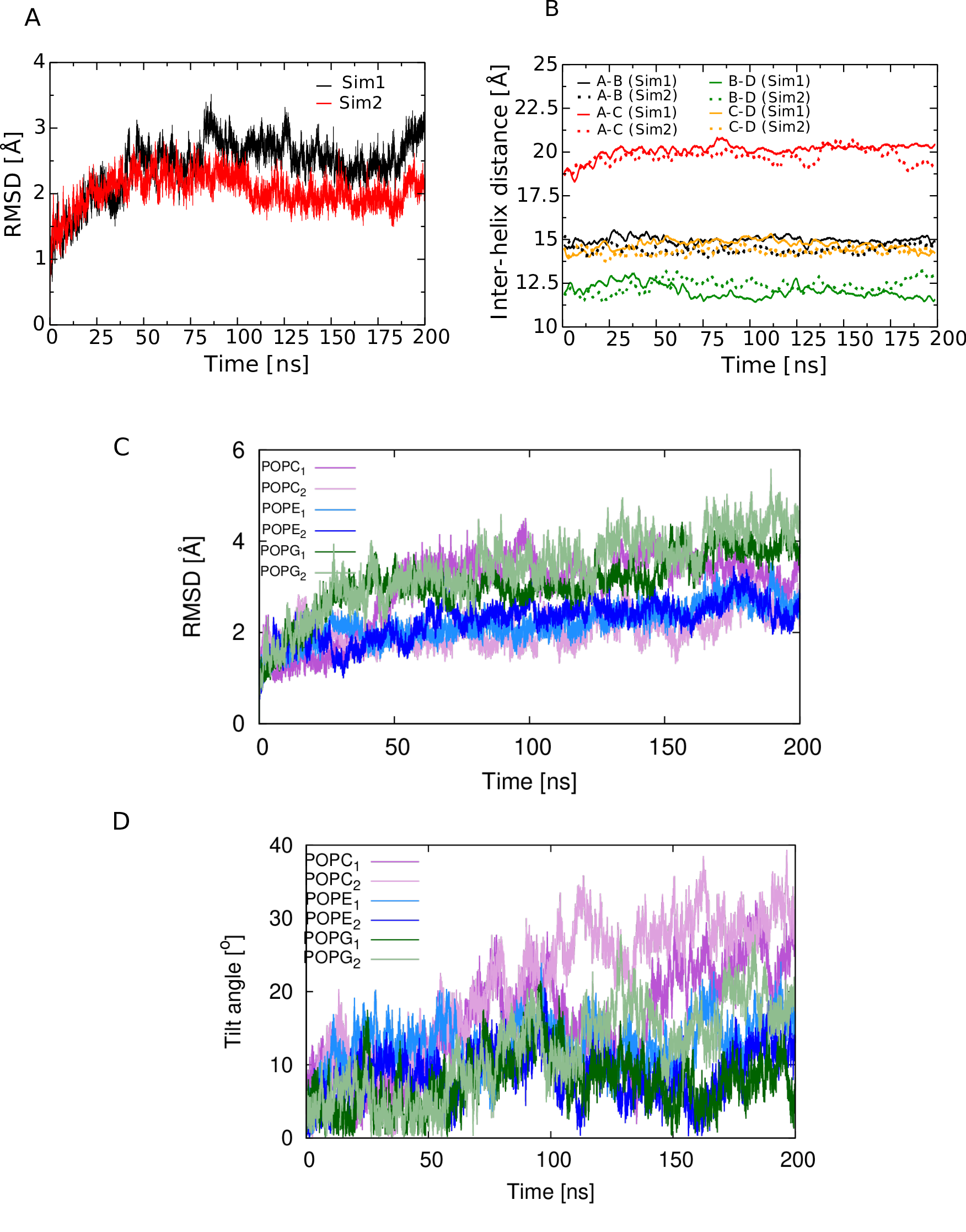


**Figure S7: – *Stability of the crystal structure of LL-37 channel in MD simulations.*** (A) RMSD values of the C_α_ atoms of the protein in the two unbiased MD simulations embedded in the POPE/POPG bilayer. After a simulation time of 40 ns, the RMSD values reach a plateau and remained stable there throughout the rest of the simulations. (B) Inter-helix distances of adjacent helix pairs of the channel (embedded in POPE/POPG bilayer) plotted over the length of the trajectories. The interhelix distance is defined as the distance between the center of mass of two adjacent helices. (C) RMSD and (D) tilt angle of LL-37 structure in homogenous lipids bilayers for two sets of simulations.


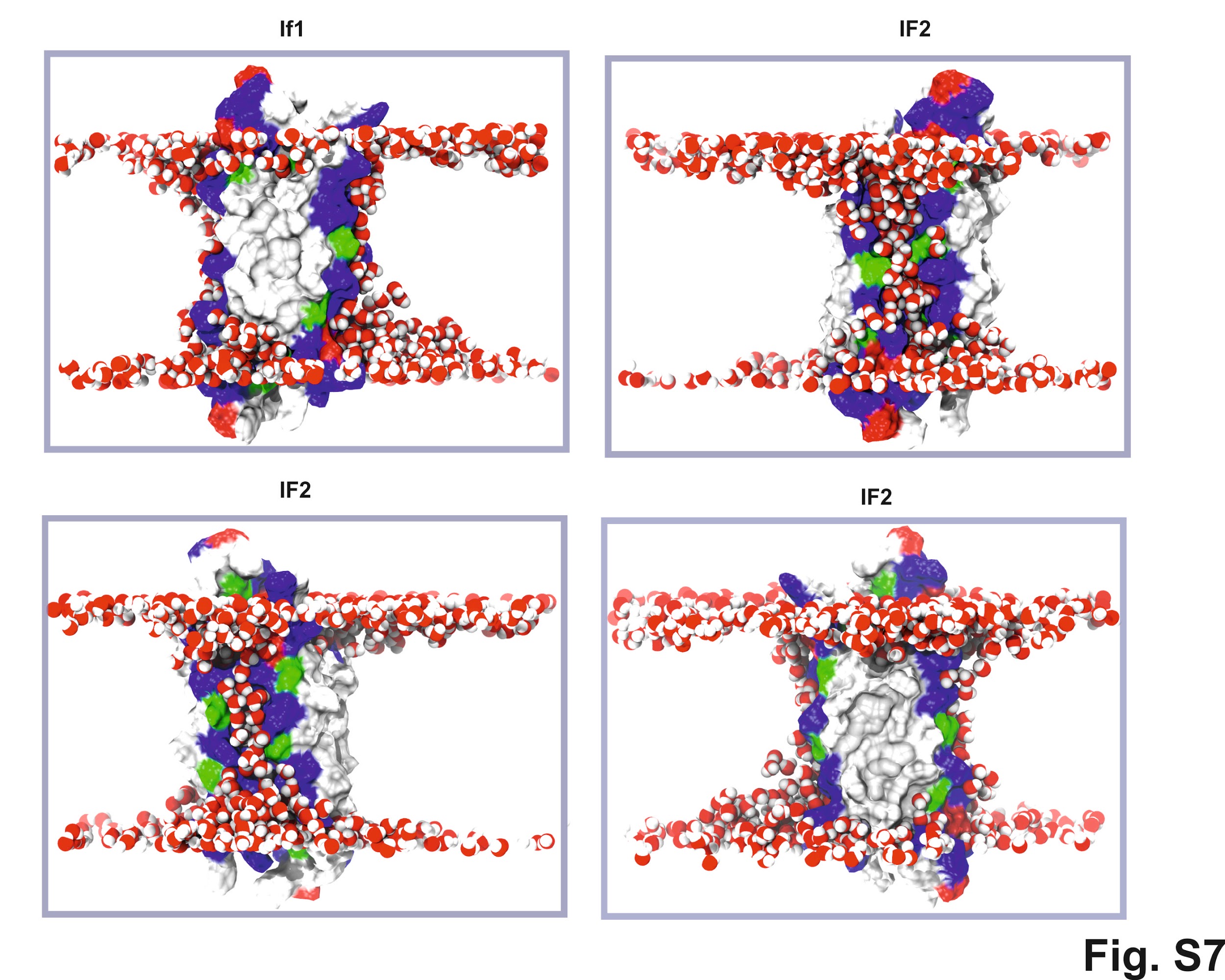


**Figure S8: – *Movement of water along the external interfaces of the channel during simulations.*** The LL-37 channel is shown in surface representation and the charged nature of residues at interfaces is highlighted with colors (blue: positive, red: negative, green: polar and white: non-polar). Water is shown in red/white spheres.


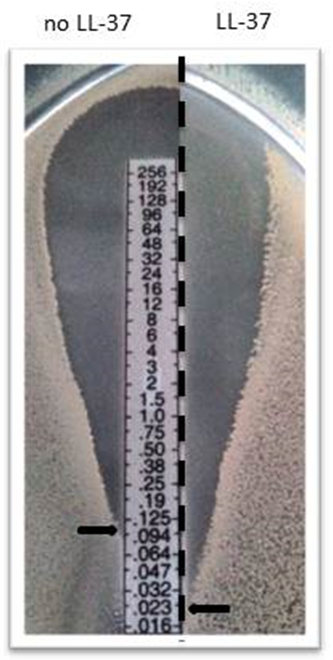


**Figure S9: – A*dditive inhibitory effect of LL-37 on plates containing daptomycin for S. aureus.*** Daptomycin E test showing susceptibility of MRSA strains between 0.016-0.023 μg/ml in the presence of LL-37 (right panel) and at 0.094 without the peptide. The image is representative of an experiment and was performed in triplicate resulting in the same susceptibility values.


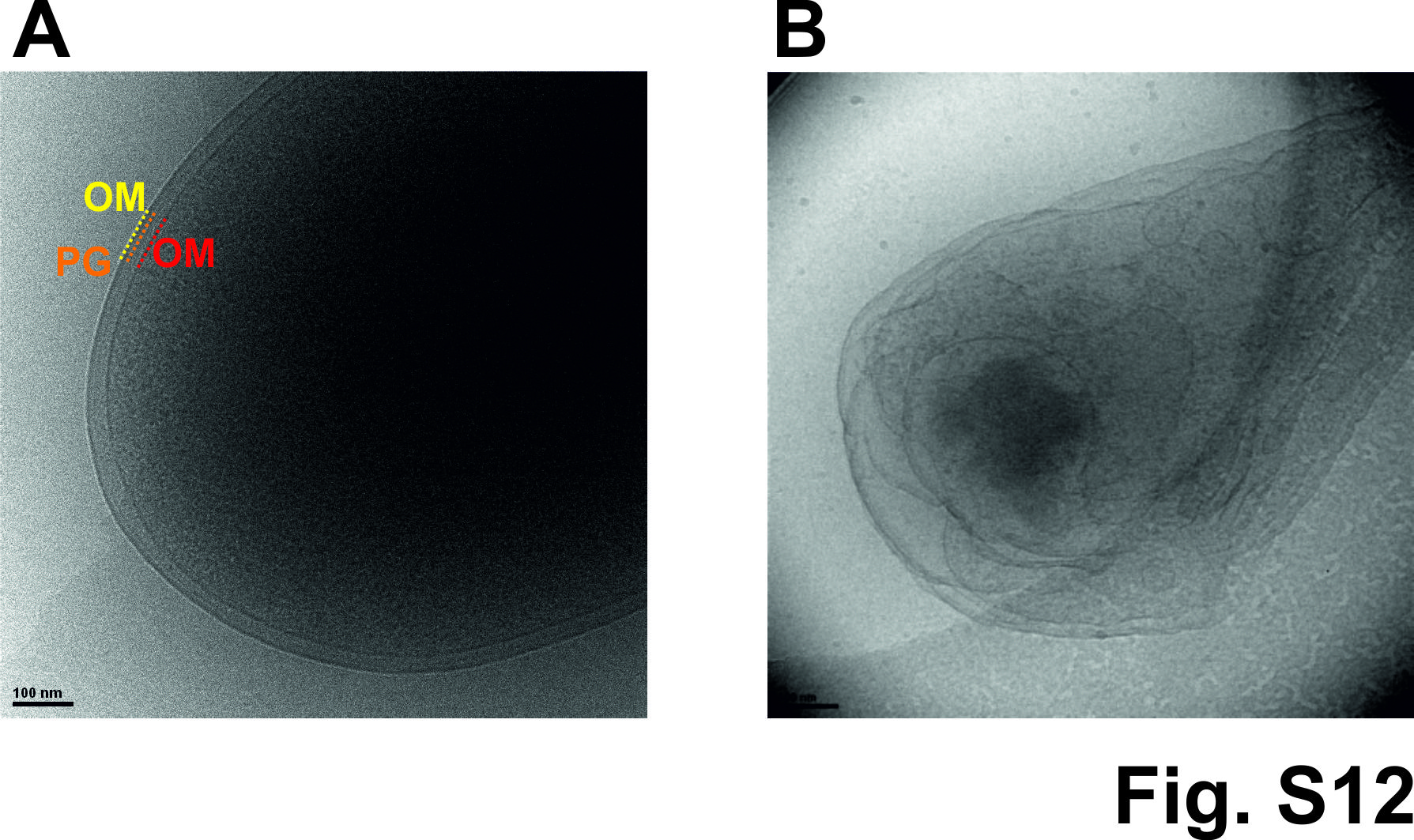


**Figure S10: –**  ***Electron micrographs of E. coli cells before and after incubation with LL-37.*** Electron microscopy of wildtype *E. coli* in the absence of peptides as control (A) shows the inner and outer membrane and peptidoglycan layer intact and clearly distinguishable. (B) Incubation of *E. coli* with LL-37 at 25 μM overnight shows the complete destruction of the cell and the remaining membrane envelopes.


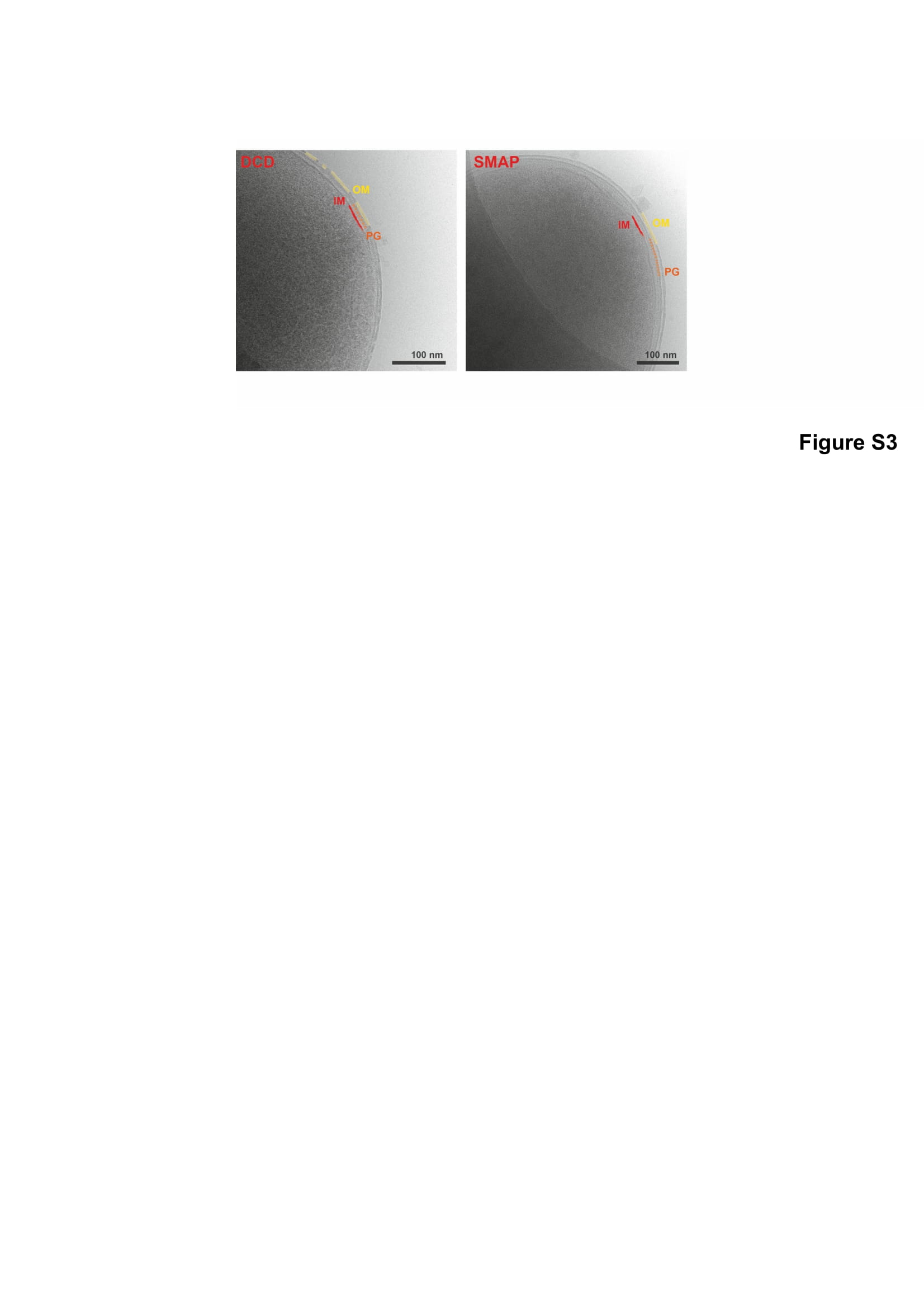


**Figure S11: –**  ***Cryo-EM pictures of LL-37 exposed to different antimicrobial peptides.*** *E. coli* cells were treated with two different antimicrobial peptides at the same concentrations of 25 μM. *E. coli* cells treated with the negatively charged human Dermcidin peptide (DCD) shows that the outer membrane remains mainly intact while pore formation in the inner membrane is visible. Sheep derived SMAP-29 carries a strong overall positive charge similar to LL-37 and shows a different mechanism of cell wall perturbation than LL-37 and DCD leaving the outer membrane largely intact while the inner membrane is significantly disturbed.


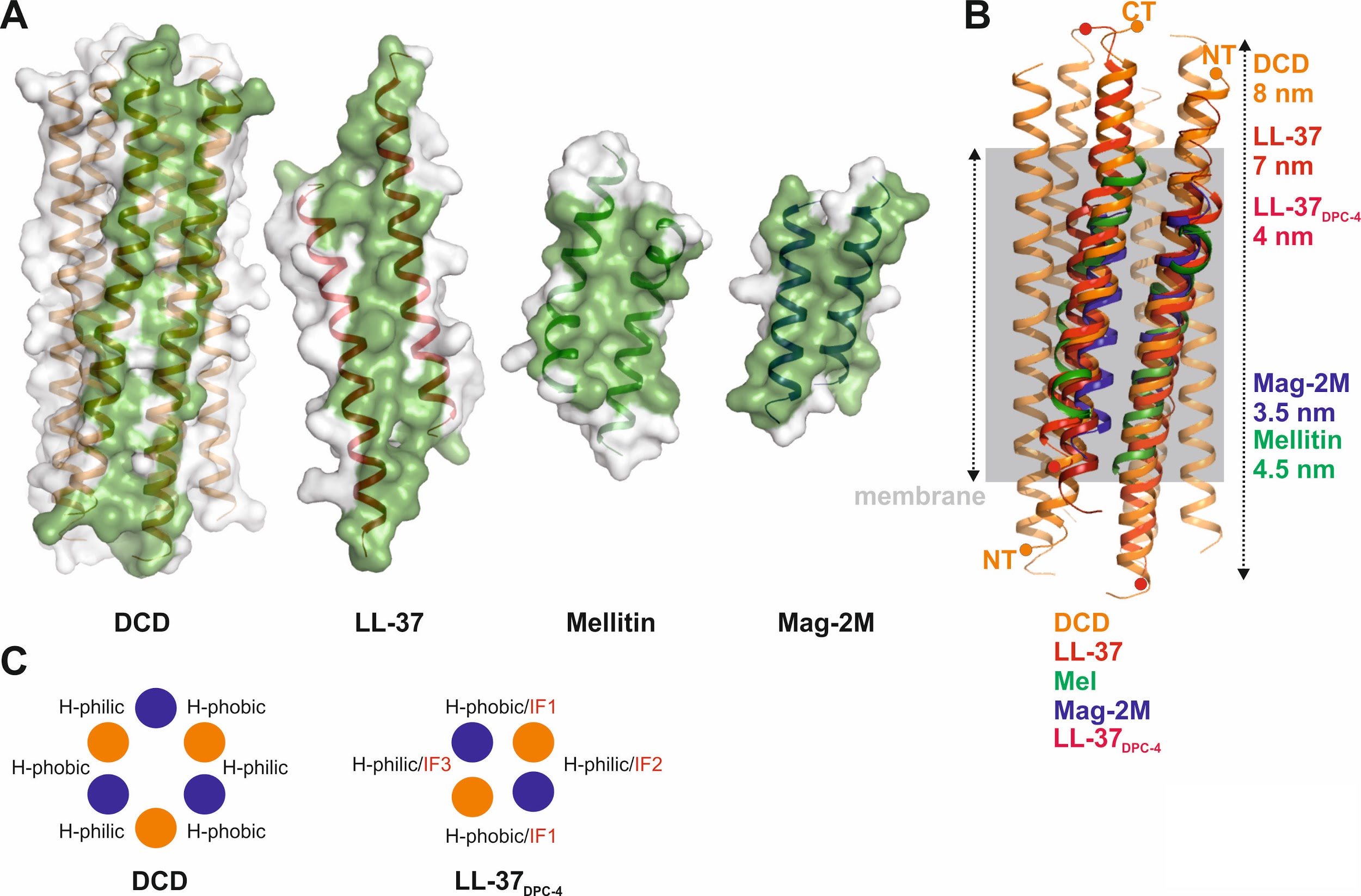


**Figure S12: – *Structural analogies between antimicrobial peptides.*** (A) Crystal structures of DCD, LL-37, Melittin, and Mag-2M shown in surface representation with hydrophobic residues colored in green. All structures show two differently structured surfaces with hydrophobic residues on one and hydrophilic residues on the opposite side. The structures are shown at the same length scale. (B) Superposition of the four structures demonstrates their structural similarity and the dimension. While DCD and LL-37 show an extension of about 7-8 nm and clearly extend a biological membrane, the other peptides (Melittin, Mag-2M and LL-37_DPC-4_) are significantly shorter with extensions of 3.5-4.5 nm. (C) Structural principles of peptide channel organization in comparison: hexameric DCD and tetrameric LL-37_DPC-4_ show alternating hydrophobic-hydrophilic interfaces.


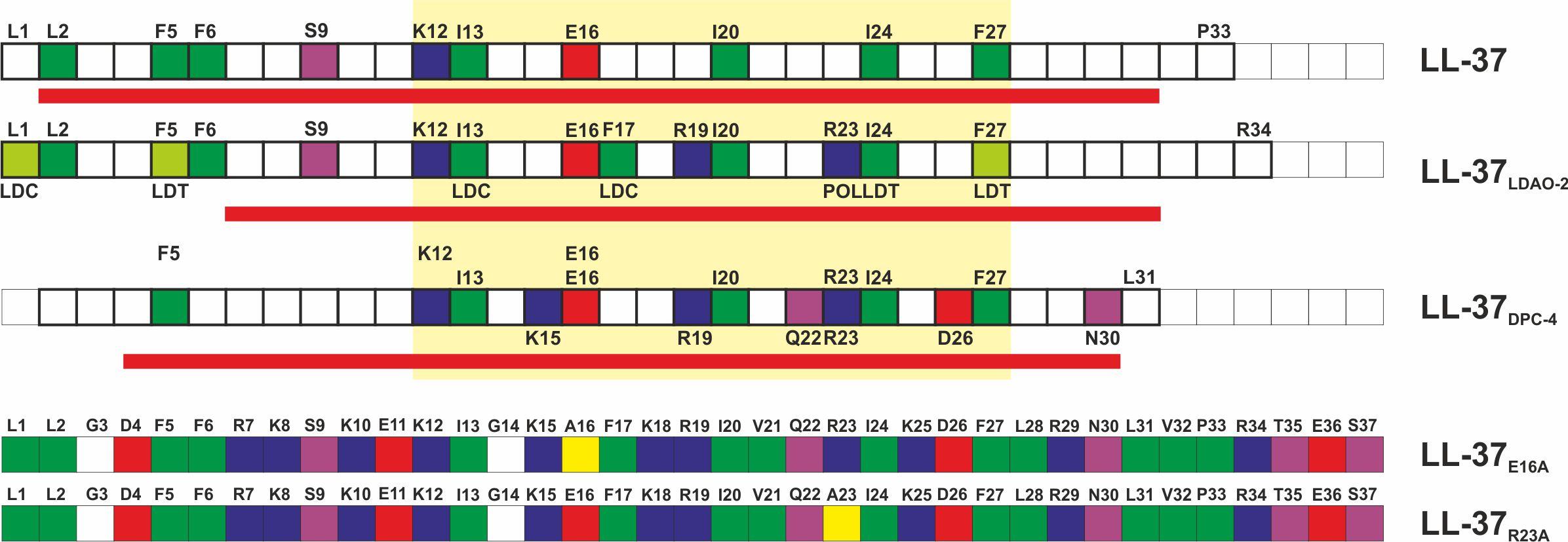


**Figure S13 - *Analysis of peptide interfaces in the crystal structures and comparison with E16A and R23A mutants.*** Residues involved in peptide-peptide interactions of the structures LL-37, LL-37_LDAO-2_ and LL-37_DPC-4_ are highlighted with colors (green **–** hydrophobic contacts; red/blue/magenta **–** charged or H-bonded contacts). The central region of LL-37 is marked in light yellow shows is the area which is important for contacts in all structures. Mutations in the sequence are marked in yellow.

**
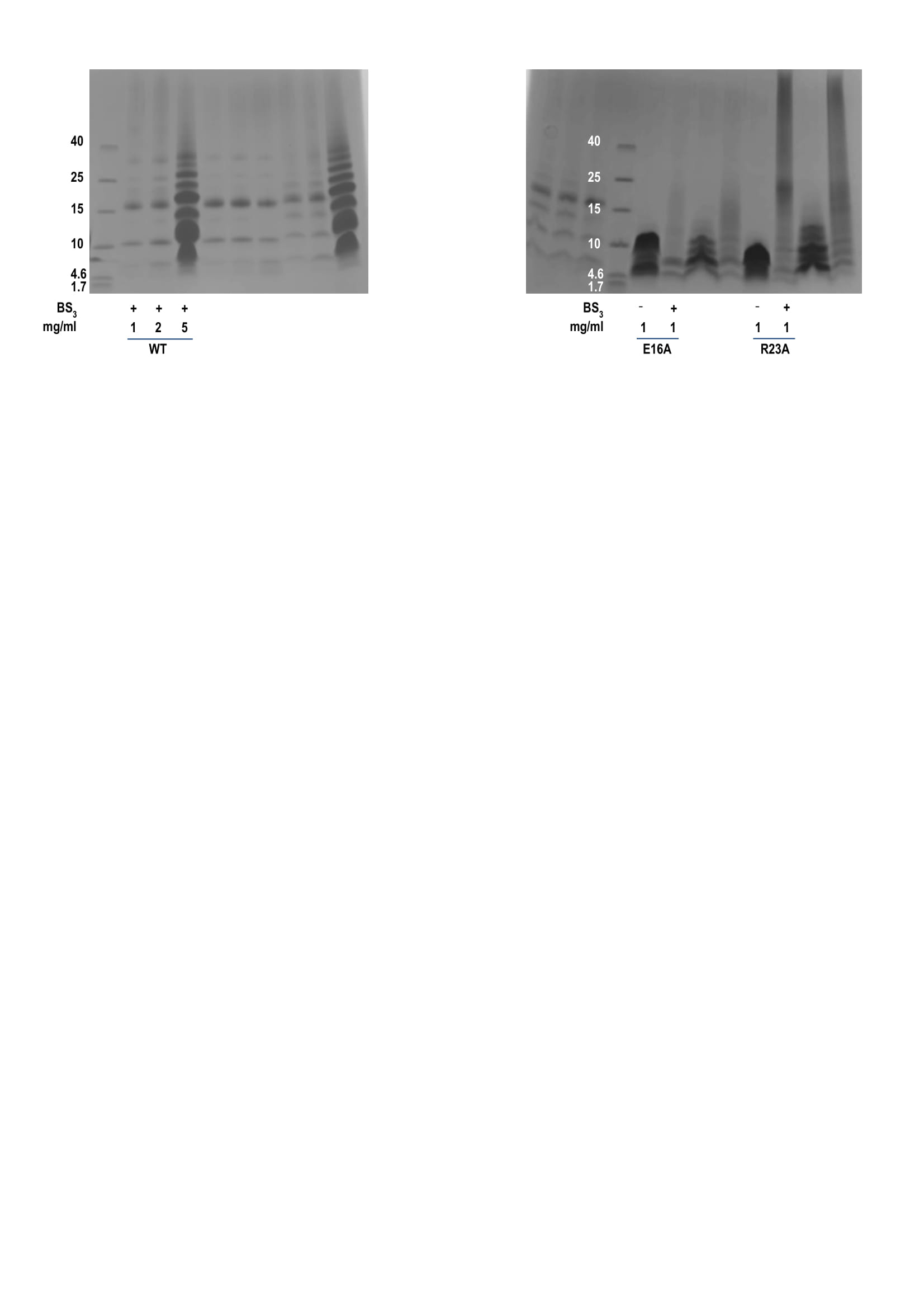
Figure S14: – *Full-length gels from cross-linking of WT (left) and E16A and R23A (right) from Figure S5B.***

**Table SI**

Antimicrobial activity testing of wild-type LL-37, E16A and R23A against *Escherichia coli K12, Staphylococcus aureus JE2, Pseudomonas syringae* and *Streptococcus pyogenes*.


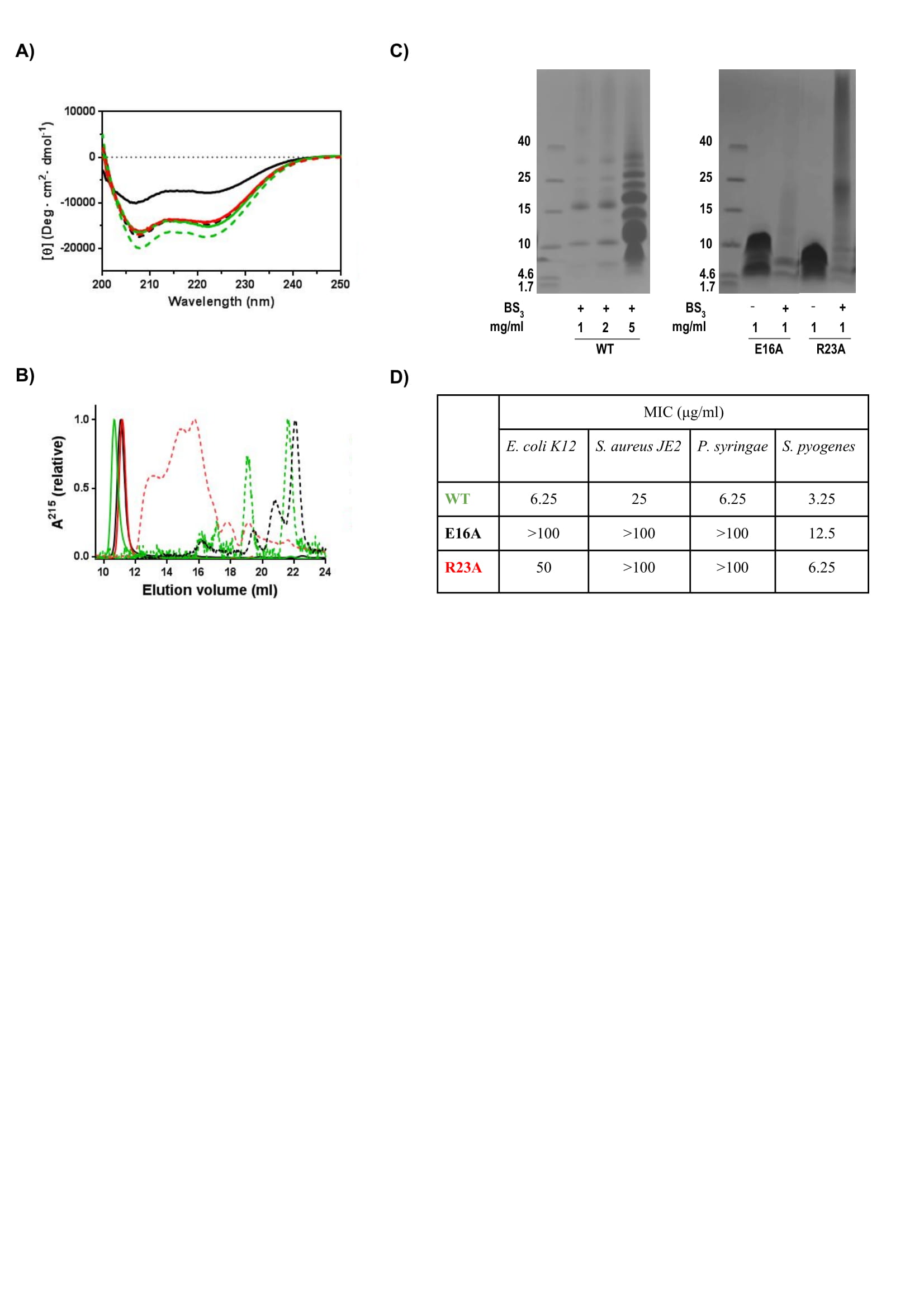


**Table SII**

Data collection and refinement statistics


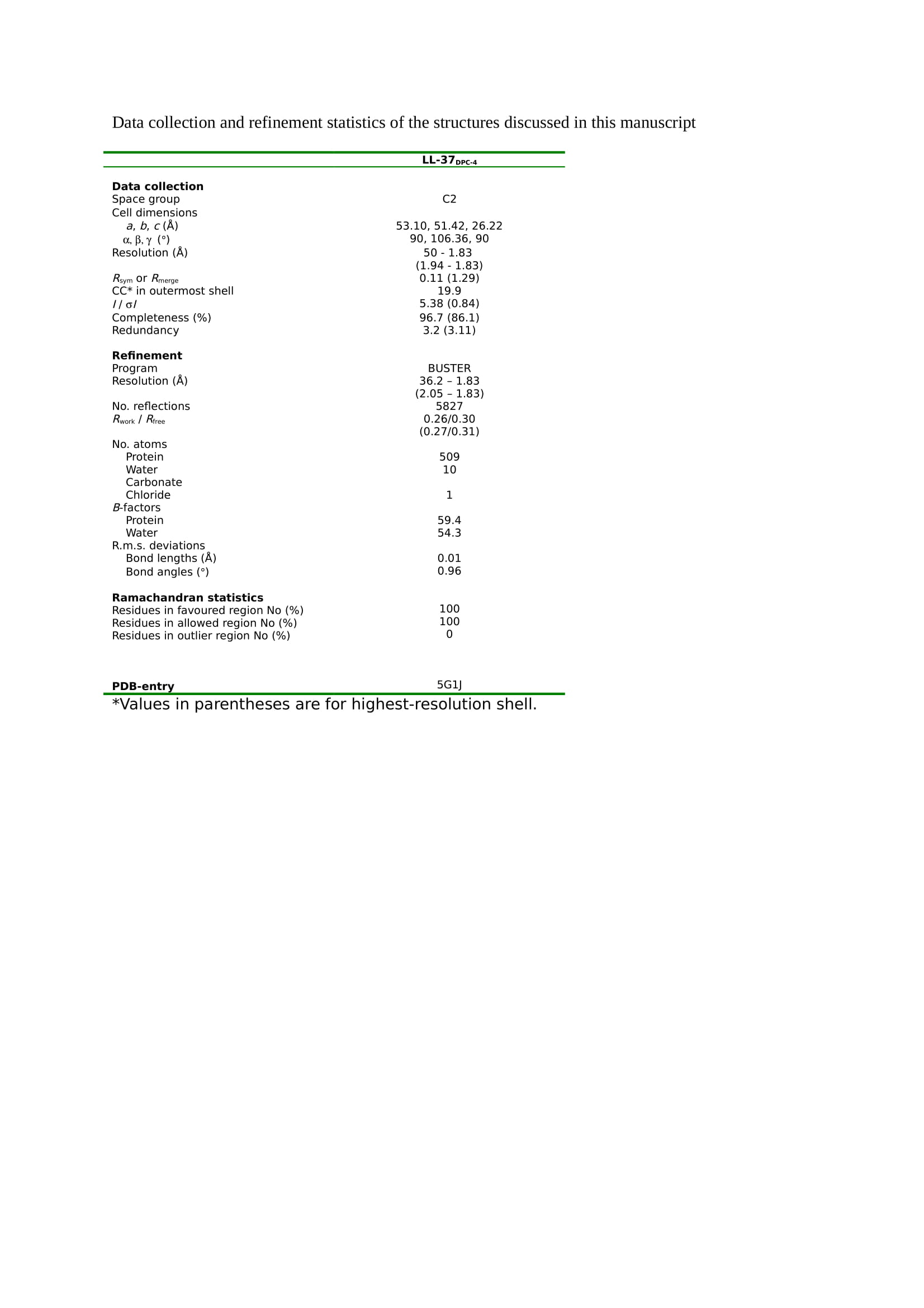


*Values in parentheses are for the highest-resolution shell.
